# Adaptive gene introgression after secondary contact

**DOI:** 10.1007/s00285-014-0802-y

**Published:** 2014-07-04

**Authors:** Hildegard Uecker, Derek Setter, Joachim Hermisson

**Affiliations:** 1 Mathematics and Biosciences Group, Faculty of Mathematics and Max F. Perutz Laboratories, University of Vienna, 1090 Vienna, Austria; 2 IST Austria, Am Campus 1, 3400 Klosterneuburg, Austria; 3 Mathematics and Biosciences Group, Faculty of Mathematics and Vienna School of Population Genetics, University of Vienna, 1090 Vienna, Austria

**Keywords:** Branching processes, Gene introgression, Adaptation, Hybridization, Genetic hitchhiking, 60J85, 92D15

## Abstract

By hybridization and backcrossing, alleles can surmount species boundaries and be incorporated into the genome of a related species. This introgression of genes is of particular evolutionary relevance if it involves the transfer of adaptations between populations. However, any beneficial allele will typically be associated with other alien alleles that are often deleterious and hamper the introgression process. In order to describe the introgression of an adaptive allele, we set up a stochastic model with an explicit genetic makeup of linked and unlinked deleterious alleles. Based on the theory of reducible multitype branching processes, we derive a recursive expression for the establishment probability of the beneficial allele after a single hybridization event. We furthermore study the probability that slightly deleterious alleles hitchhike to fixation. The key to the analysis is a split of the process into a stochastic phase in which the advantageous alleles establishes and a deterministic phase in which it sweeps to fixation. We thereafter apply the theory to a set of biologically relevant scenarios such as introgression in the presence of many unlinked or few closely linked deleterious alleles. A comparison to computer simulations shows that the approximations work well over a large parameter range.

## Introduction

Hybridization between related species is a common phenomenon. Indeed, Mallet (2005) estimates that at least $25\,\%$ of plant species and $10\,\%$ of animal species still interbreed. The disappearance of natural habitat barriers following environmental change, the introduction of foreign species, the escape of domesticated animals into the wild, and the cultivation of crops all create new regions of species range overlap and consequently cause high rates of hybridization. Despite reproductive barriers, hybridization between related species is often not completely prohibited and leads to the production of viable and fertile offspring. In the course of backcrossing with a parental species, alien genetic material is lost, but some part of it may be permanently incorporated into the genome of the sister species. The introgression of genes from one species into another occurs over a wide range of taxa (Rhymer and Simberloff 1996; Lindner et al. 1998; Arnold et al. 1999; Arnold 2004; Miller et al. 2012; Ellstrand et al. 2013). The introgression of genes from feral to wild animals (Adams et al. 2003; Beaumont et al. 2001; Gottelli et al. 1994; Rhymer and Simberloff 1996) or from introduced to native species (Rhymer and Simberloff 1996; Fitzpatrick et al. 2010) poses ecological risks and if extensive may entail a loss of biodiversity.

In addition, evidence for the transfer of adaptations across species boundaries is growing (Arnold et al. 1999; Arnold 2004; Whitney et al. 2006; Schwenk et al. 2008; Arnold and Martin 2009; The *Heliconius* Genome Consortium 2012; Hedrick 2013). Introgression of genes can hence take direct influence on the evolutionary routes of a species and speed up adaptation. For example, the introduced sunflower species *Helianthus annuus* likely has acquired resistance genes from the native and locally adapted species *H. debilis* in Texas, allowing it to expand its species range southwards (Heiser 1951; Whitney et al. 2006). Similarly, Abi-Rached et al. (2011) suggest that positively selected immune system alleles from Neanderthals and Denisovans might have introgressed into modern humans. In agriculture, adaptive gene introgression can potentially constitute a major risk: adaptive herbivore, insecticide, or pathogen resistance genes from (possibly genetically modified) crops can spread to wild relatives, severely complicating weed control (Snow 2002; Snow et al. 2003; Stewart et al. 2003). Importantly, Snow et al. (2003) show that a transgene can reduce herbivory and increase fitness in a wild sunflower under natural conditions.

Early-generation hybrids, even if not entirely infertile or inviable, frequently suffer from strongly reduced fitness. Often, hybrids display an intermediate phenotype that is maladapted to either parental niche. The low hybrid fitness can also result from genetic incompatibilities. By backcrossing with one of the parental species, alleles that prove to be deleterious in the foreign genetic background or cause maladaptation to the parental niches can be purged and fitness restored (Heiser 1951; Arnold et al. 1999). The probability of successful gene introgression critically depends on the strength of this fitness bottleneck.

Theoretical models on adaptive gene introgression that take a reduction in hybrid fitness into account usually assume that a pre-defined number of backcrosses are required in order to lose the deleterious material and obtain a positively selected type (Demon et al. 2007; Gosh and Haccou 2010; Gosh et al. 2012a, b). This assumes that the deleterious effects are homogeneously spread over the genome of a diploid organism and that an appreciable amount of deleterious alleles is required to have a measurably impact on fitness. Focusing on other aspects of gene introgression, such as the impact of a temporally varying environment (Gosh et al. 2012b) or life history traits (Demon et al. 2007), these models greatly simplify the underlying genetics. A step towards more realistic population genetic models has been made by Gosh et al. (2012a). Their analysis remains, however, restricted to the most basic scenario in which a single deleterious allele is linked to the locus under positive selection (see also Barton 1979). For a neutral marker locus, in contrast, the impact of a genetic barrier consisting of an arbitrary number of deleterious alleles has been investigated (Bengtsson 1985; Barton and Bengtsson 1986).

In this paper, we focus on a single hybridization event and examine the impact of linked and unlinked deleterious alleles on the introgression process of an adaptive allele. The deleterious effect of alleles is caused by maladaptation to the new environment and is independent of the genetic background. We first set up a Moran-like model which describes the evolution of the population by genetic drift, selection, and recombination. In the first part of the model analysis, we apply the theory of reducible multitype branching processes to determine by how much deleterious alleles reduce the introgression probability of a favorable allele in dependence of the strength of selection and linkage. The second part considers the probability that closely linked deleterious alleles “hitchhike” to fixation. The analysis relies on a separation of the process into a strongly stochastic phase, in which a haplotype carrying the beneficial allele establishes, and a deterministic phase, in which it sweeps through the population, possibly losing deleterious material by recombination with wildtype individuals. These recombination events and the subsequent establishment or loss of haplotypes with fewer deleterious alleles are again subject to strong stochasticity. In this analysis, we again resort to the theory of branching processes. The derived approximations are applied to a variety of biological scenarios and complemented by computer simulations. We close the paper with a discussion.

## Full model and simulations

We consider a large population of $N$ haploid individuals. The theory also applies to diploids without dominance if we can assume Hardy-Weinberg equilibrium, but we use the haploid formalism throughout the paper. Through a single hybridization event, a hybrid individual is introduced to the population (for diploids, the alien alleles arrive in the foreign habitat at the haploid stage, i.e., for plants, by pollen dispersal). The hybrid carries an adaptive allele as well as a number of deleterious alleles. Deleterious alleles are either physically linked to the adaptive allele or unlinked. We assume that this initial hybrid haplotype carries $I$ and $J$ linked deleterious alleles to the left and the right of the beneficial allele, respectively, and $F$ unlinked alleles. By recombination with wildtype individuals, haplotypes with fewer introgressed alleles can be generated, leading to a hybrid swarm. Selection on the deleterious alleles relies on maladaptation to the environment and is independent of the genomic context. We assume that all wildtype individuals have the same fitness and introgressed alleles interact identically with all wildtype backgrounds. The fitness of an individual is thus fully determined by the introgressed alleles that it carries.

The evolution of the population is described by the following scheme, which represents a Moran model with recombination: At rate $N$, two individuals are chosen to reproduce and generate a single offspring. During reproduction, recombination can take place. In order to simplify our bookkeeping of genotypes in the analytical treatment, we restrict ourselves to single crossover among the linked alleles. Multiple crossover is unlikely to happen over recombination distances $r$ with $r^2\ll r$ so that the model captures scenarios of tight linkage. Considering larger recombination distances (where multiple crossover gets likely) or gene conversion requires a straightforward extension of the formalism, in which a larger number of genotypes can be generated by recombination with wildtype individuals. Unlinked alleles are inherited with probability one-half. The offspring replaces an individual that is chosen based on its fitness. For notational simplicity, we assign the numbers $1$ to $N$ to the individuals. Individual number $k$ is then chosen with probability $\frac{1-\sigma ^{(k)}}{\sum \nolimits _{i=1}^N (1-\sigma ^{(i)})}$, where $\sigma ^{(k)}=0$ for wildtype individuals. I.e., $\sigma ^{(k)}$ is the Malthusian fitness of individual $k$ in a wildtype population. All three individuals that are involved in a reproduction event are chosen with replacement, i.e., the same individual might be chosen twice.

The simulation program implements the successive events without consideration for the time spans between them. As we are only interested in probabilities, this does not influence the results. The number of replicates is chosen so that error bars vanish in the symbols in all plots. The simulation program is written in the C++ programming language, making use of the *Gnu Scientific Library* (Galassi et al. 2009).

The full model does not allow for an analytical treatment. In the following sections, we therefore consider approximations to the introgression process.

## The early phase of spread

A single evolutionary step involves three individuals: two individuals that reproduce and one that dies. In a large population, as long as introgressed haplotypes are rare, it is unlikely that more than one hybrid individuals are involved in a single event (or that the same individual is involved twice). Formally, this means that terms of order $(n_{\text {intro}}/N)$ in the transition rates, where $n_{\text {intro}}$ denotes the number of individuals with introgressed alleles, are negligible. Consequently, hybrids suffer (nearly) independent fates in the early phase of spread, and the process is therefore well described by a multitype branching process. The branching process is strictly recovered in the limit $N \rightarrow \infty$.

The lack of interaction among hybrids entails that types with introgressed material only recombine with wildtype individuals. This implies that by recombination, they can only lose, not gain deleterious alleles, and we encounter a special instance of a reducible multitype branching process (cf. also Barton and Bengtsson 1986; Demon et al. 2007; Gosh et al. 2012b). By recombination, types that carry only deleterious alleles but not the beneficial allele are generated. We do not consider these types in the following (within a branching process approach, they are doomed to extinction) but focus on carriers of the advantageous allele. In the main text of the paper, we assume that all unlinked deleterious alleles have the same effect size. A generalization of the main results to arbitrary effect sizes is given in Appendix D.

**Fig. 1:**
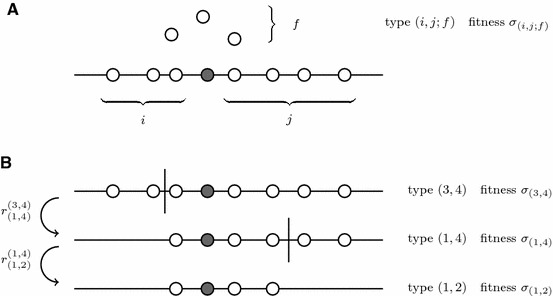
An illustration of the branching model. The *dark dot* represents the advantageous allele, the *blank dots* represent deleterious alleles. $i$ and $j$ give the number of deleterious alleles to the *left* and *right* of the advantageous allele and $f$ the number of unlinked deleterious alleles. Panel **b** illustrates how linked deleterious alleles are lost by recombination with wildtype individuals. “Fitness” refers to the Malthusian fitness

We call an individual that carries the beneficial allele with $i$ deleterious alleles to the left, $j$ deleterious alleles to the right and $f$ unlinked deleterious alleles an individual of type $(i,j;f)$ (cf. Fig. 1a). Its net selection coefficient is denoted by $\sigma _{(i,j;f)}$. We set $(i,j) \equiv (i,j;0)$. The recombinant offspring of a type $(i,j)$ individual are either of type $(i,k)$ with $k < j$ or of type $(k,j)$ with $k< i$. Such recombination events may happen with probabilities $r^{(i,j)}_{(i,k)}$ and $r^{(i,j)}_{(k,j)}$, respectively. An instance of repeated recombination events is depicted in Fig. 1b. The overall probability that a recombination event takes place is given by the sum $r_{(i,j)}=\sum \nolimits _{k=0}^{j-1} r^{(i,j)}_{(i,k)} + \sum \nolimits _{k=0}^{i-1} r^{(i,j)}_{(k,j)}$. The number of unlinked deleterious alleles that are inherited by an offspring individual is binomially distributed with parameter $0.5$. We obtain for the per capita transition rates of the possible events in the branching process:

Death

1a $$\begin{aligned} P((i,j;f) \rightarrow \emptyset ) =1-\sigma _{(i,\,j;\,f)}, \end{aligned}$$

Birth—with coupled recombination

1b $$\begin{aligned} P((i,j;f) \rightarrow \{(i,j;f),(i,j;g)\})&= \left( {\begin{array}{c}f\\ g\end{array}}\right) \left( \frac{1}{2}\right) ^f (1-r_{(i,j)}),\nonumber \\ P((i,j;f) \rightarrow \{(i,j;f),(i,k;g)\})&= \left( {\begin{array}{c}f\\ g\end{array}}\right) \left( \frac{1}{2}\right) ^f r^{(i,j)}_{(i,k)} \quad \text {for} \quad k<j,\nonumber \\ P((i,j;f) \rightarrow \left\{ (i,j;f),(k,j;g)\right\} )&= \left( {\begin{array}{c}f\\ g\end{array}}\right) \left( \frac{1}{2}\right) ^f r^{(i,j)}_{(k,j)} \quad \text {for} \quad k<i. \end{aligned}$$

## The probability of adaptive gene introgression

First, we focus on the probability that the beneficial allele establishes in the population. Once the beneficial allele is sufficiently frequent, it is very unlikely to be lost again. The extinction probability of the branching process as described in the previous section is thus a good approximation for the extinction probability of the beneficial allele in the full model (and the introgression probability is the complementary probability). We denote by $Q_{(i,j;f)}$ the extinction probability of the process that is initiated by exactly one individual of type $(i,j;f)$.

### **Theorem 1**

The extinction probability $Q_{(I,J;F)}$ can be calculated by recursively solving the system of quadratic equations

2 $$\begin{aligned}&(1-r_{(i,j)})\left( \frac{1}{2}\right) ^f Q_{(i,j;f)}^2\nonumber \\&\quad + \sum \limits _{g=0}^{f} \left( {\begin{array}{c}f\\ g\end{array}}\right) \left( \frac{1}{2}\right) ^f \left\{ \sum \limits _{k=0}^{i-1} r^{(i,j)}_{(k,j)}Q_{(k,j;g)} + \sum \limits _{k=0}^{j-1} r^{(i,j)}_{(i,k)}Q_{(i,k;g)} \right\} Q_{(i,j;f)}\nonumber \\&\quad + \sum \limits _{g=0}^{f-1} \left( {\begin{array}{c}f\\ g\end{array}}\right) \left( \frac{1}{2}\right) ^f (1-r_{(i,j)}) Q_{(i,j;g)}Q_{(i,j;f)}\nonumber \\&\quad -(2-\sigma _{(i,j;f)})Q_{(i,j;f)} +1-\sigma _{(i,j;f)}=0, \nonumber \\&\quad i \in \{0,\dots ,I\}, \quad j \in \{0,\dots ,J\}, \quad f \in \{0,\dots ,F\}, \end{aligned}$$

where always the smaller root of the equation has to be used.

We only give an illustrative derivation of Eq. (2) here and move the full proof to Appendix A.

Consider a branching process initiated by an individual of type $(i,j;f)$. With probability $\frac{1-\sigma _{(i,j;f)}}{2-\sigma _{(i,j;f)}}$, the founding individual dies before it reproduces, in which case the lineage is immediately extinct. With probability $$\left( {\begin{array}{c}f\\ g\end{array}}\right) \left( \frac{1}{2}\right) ^f\frac{1-r_{(i,j)}}{2-\sigma _{(i,j;f)}}$$, it reproduces and generates a non-recombinant offspring with $g$ unlinked alleles, i.e., an offspring of type $(i,j;g)$. With probability $$\left( {\begin{array}{c}f\\ g\end{array}}\right) \left( \frac{1}{2}\right) ^f\frac{r^{(i,j)}_{(i,k)}}{2-\sigma _{(i,j)}}$$ (or $$\left( {\begin{array}{c}f\\ g\end{array}}\right) \left( \frac{1}{2}\right) ^f\frac{r^{(i,j)}_{(k,j)}}{2-\sigma _{(i,j)}}$$), it reproduces and gives birth to a type $(i,k;g)$ (or $(k,j;g)$) individual with $0\le k j$ (or $0\le k < i$). After reproduction, there are now two individuals, each of which is again the founding individual of a lineage. In order for the original lineage to go extinct, both of these lineages have to die out. It therefore holds for the extinction probability $Q_{(i,j;f)}$:

3 $$\begin{aligned} Q_{(i,j;f)}&= \frac{1-\sigma _{(i,j;f)}}{2-\sigma _{(i,j;f)}} + \sum \limits _{g=0}^{f} \left( {\begin{array}{c}f\\ g\end{array}}\right) \left( \frac{1}{2}\right) ^f \frac{1-r_{(i,j)}}{2-\sigma _{(i,j;f)}} Q_{(i,j;g)}Q_{(i,j;f)}\nonumber \\&+ \sum \limits _{g=0}^{f} \left( {\begin{array}{c}f\\ g\end{array}}\right) \left( \frac{1}{2}\right) ^f \left\{ \sum \limits _{k=0}^{i-1} \frac{r^{(i,j)}_{(k,j)}}{2\!-\!\sigma _{(i,j;f)}} Q_{(k,j;g)} \!+\! \sum \limits _{k=0}^{j-1} \frac{r^{(i,j)}_{(i,k)}}{2\!-\!\sigma _{(i,j;f)}} Q_{(i,k;g)} \right\} Q_{(i,j;f)}.\nonumber \\ \end{aligned}$$

By rearranging terms, we obtain Eq. (2).

For the special case $F=0$, Eq. (2) simplifies to

4 $$\begin{aligned} 0&= (1-r_{(i,j)}) Q_{(i,j)}^2 + \sum \limits _{k=0}^{j-1} r^{(i,j)}_{(i,k)} Q_{(i,j)}Q_{(i,k)} + \sum \limits _{k=0}^{i-1} r^{(i,j)}_{(k,j)} Q_{(i,j)}Q_{(k,j)} \nonumber \\&+ 1-\sigma _{(i,j)} - (2-\sigma _{(i,j)}) Q_{(i,j)}, \quad i\in \{0,\dots ,I\}, \quad j\in \{0,\dots ,J\}.\quad \end{aligned}$$

If all deleterious alleles are unlinked (i.e., $I=J=0$; abbreviate $(0,0;g)\equiv g$), Eq. (2) yields:

5 $$\begin{aligned} 0 \!= \!\left( \frac{1}{2}\right) ^f Q_f^2 \!+\! \left( \frac{1}{2}\right) ^f \sum \limits _{g=0}^{f-1} \left( {\begin{array}{c}f\\ g\end{array}}\right) Q_g Q_f + 1\!-\!\sigma _f \!-\!(2-\sigma _f) Q_f, \quad f \in \{0,\dots ,F\}.\nonumber \\ \end{aligned}$$

Implications of these results are discussed in Sect. 6.

In Appendix B, we relate our results to results by Bengtsson (1985) and Barton and Bengtsson (1986) on the impact of a cline on the spread of a neutral marker allele. Our results are consistent with Bengtsson (1985) and Barton and Bengtsson (1986) when all loci are only loosely linked or unlinked but deviate for tight linkage of the deleterious allele.

## The hitchhiking probability

### General idea

If the effects of closely linked deleterious alleles are not too harmful, namely if $\sigma _{(i,j)}-r_{(i,j)}>0$, the beneficial allele can drag (some of) these deleterious alleles along to fixation. In this section, we develop a framework for determining the hitchhiking probabilities conditioned on fixation of the beneficial allele. The approach is based on a split of the process into two phases. After the original hybridization event, the beneficial allele must establish in the population. We call this the “stochastic phase”. In the previous section, we were concerned with the establishment probability itself. Here, we further derive which haplotype $(i,j)$ will escape stochastic loss in this initial phase conditioned on survival of the process. We assume that only one haplotype escapes. This is a very likely outcome of the stochastic phase under many circumstances because the establishment probability of each type is low. Since this establishment happens while the introgressed types are rare, we base the derivation on the multitype branching process as before. Once established, type $(i,j)$ increases in frequency approximately as predicted by deterministic growth. If no further recombination events were to happen, it would rise to fixation following the logistic equation

6 $$\begin{aligned} \dot{x}_{(i,j)}(t) = \sigma _{(i,j)} x_{(i,j)}(t)(1-x_{(i,j)}(t)). \end{aligned}$$

However, during the sweep, types with fewer deleterious alleles can still be generated by recombination. If one of these types establishes, it outcompetes type $(i,j)$. Building on theory by Hartfield and Otto (2011), we describe the production and possible establishment of these types by a time-inhomogeneous branching process with immigration. Although the generation and establishment of new haplotypes is subject to strong stochasticity, we refer to this phase as to the “deterministic phase” because we model the frequency paths of the established haplotypes deterministically.

We first give a derivation for the case without unlinked deleterious material ($F=0$), and subsequently generalize the approximation to $F>0$.

### The stochastic phase

As a first step, we determine which haplotype “rescues” the introgression process given that the process does not go extinct. For this initial phase, we again resort to the multitype branching process as defined in Eq. (1). As before, the process is initiated by a single individual of type $(I,J)$. If $\sigma _{(I,J)}-r_{(I,J)}>0$, type $(I,J)$ has the chance to establish a permanent lineage of its own type. If $\sigma _{(I,J)}-r_{(I,J)}\le 0$, type $(I,J)$ itself will go extinct with probability $1$ (ignoring the possibility of fixation by drift). However, until extinction, recombinant offspring with fewer deleterious alleles can be generated and rescue the process. In that case, to determine the “rescue type”, we can consider all recombination pathways that lead to establishment of the beneficial allele and determine with which (relative) probability the various paths are realized. This idea is key for the derivation of the approximation in this section.

Throughout the analysis, the total number of recombination events from type $(I,J)$ to any other type until extinction of type $(I,J)$ constitutes a central quantity. This follows Serra (2006) and Serra and Haccou (2007). For $\sigma _{(I,J)}-r_{(I,J)}<0$, we denote the corresponding probability generating function (p.g.f.) by $h(s)$. For $\sigma _{(I,J)}-r_{(I,J)}>0$ and extinction of type $(I,J)$, we consider the number of recombination events conditioned on extinction of type $(I,J)$ and denote the p.g.f. by $\hat{h}(s)$. $h(s)$ and $\hat{h}(s)$ can be explicitly calculated for our model and are given by Lemma 6 in Appendix E.

As a first step, we derive an alternative expression for the survival probability of the process. To do so, we group the recombinant offspring of type $(I,J)$ individuals into two classes: (1) individuals that found processes that survive (2) individuals that found processes that go extinct. We denote by $Y_{+}$ and $Y_{-}$ the random number of recombination events from type $(I,J)$ to type $1$ and type $2$ individuals, respectively. In the lemma, we rewrite the survival probability of the process in terms of the expected number of successful recombinant lineages and an error term.

#### **Lemma 1**

Let $\sigma _{(I,J)}-r_{(I,J)} < 0$. The survival probability of the process can be written as

7 $$\begin{aligned} 1-Q_{(I,J)} = \left( \sum _{k=0}^{I-1} r_{(k,J)}^{(I,J)} (1-Q_{(k,J)}) + \sum _{k=0}^{J-1} r_{(I,k)}^{(I,J)}(1-Q_{(I,k)})\right) \frac{\frac{\mathrm {d}}{\mathrm {d}s}h(s)|_{s=1}}{r_{(I,J)}}-R_1,\nonumber \\ \end{aligned}$$

where the error term $R_1$ is given by

8 $$\begin{aligned} R_1= \frac{\partial }{\partial s_0} \left( \frac{h_1(s_0,s_1)-h_1(0,s_1)}{s_0}\right) \bigg |_{s_0=s_1=1} \end{aligned}$$

with

9 $$\begin{aligned} h_1(s_0,s_1) = h(P_\mathrm{success}s_0 +(1-P_\mathrm{success})s_1) \end{aligned}$$

and

10 $$\begin{aligned} P_\mathrm{success} = \frac{\sum _{k=0}^{I-1} r_{(k,J)}^{(I,J)}(1-Q_{(k,J)}) + \sum _{k=0}^{J-1} r_{(I,k)}^{(I,J)}(1-Q_{(I,k)})}{r_{(I,J)}}. \end{aligned}$$

#### *Proof*

A recombinant offspring of a type $(I,J)$ individual founds an infinite lineage with probability $P_{\text {success}}$ and a lineage that goes extinct with probability $1-P_{\text {success}}$. According to Lemma 7 in Appendix E, the joint p.g.f of $Y_+$ and $Y_-$ is given by

11 $$\begin{aligned} h_1(s_0,s_1) = h(P_{\text {success}}s_0+ (1-P_{\text {success}})s_1), \end{aligned}$$

and we obtain for the expected number of type $1$ individuals:

12 $$\begin{aligned} E[Y_+] = \frac{\partial }{\partial s_0} h_1(s_0,s_1)|_{s_0=s_1=1} =P_{\text {success}} \frac{\mathrm {d}}{\mathrm {d}s}h(s)|_{s=1}. \end{aligned}$$

Now note:

13 $$\begin{aligned} 1-Q_{(I,J)} = P(Y_+>0) = E[Y_+] - R_1 \end{aligned}$$

with

14 $$\begin{aligned} R_1&= E[Y_+]-P(Y_+>0)=P(Y_+=2)+2P(Y_+=3)+3P(Y_+=4)+\dots \nonumber \\&= \frac{\partial }{\partial s_0} \left( \frac{h_1(s_0,s_1)-h_1(0,s_1)}{s_0}\right) \bigg |_{s_0=s_1=1}. \end{aligned}$$

I.e., if $P(Y_+>1) \approx 0$, the expected number of recombination events from type $(I,J)$ individuals to individuals with fewer mutations that found a successful lineage approximates the survival probability of the process. $\square$

#### *Remark*

For $\sigma _{(I,J)} - r_{(I,J)} > 0$, we can consider the process conditioned on extinction of type $(I,J)$. In this case, an analogous result holds if we replace $h(s)$ by $\hat{h}(s)$.

Results of a similar structure, which approximate weak recombination, appear in Iwasa et al. (2004a, Eq. (5)) and Serra and Haccou (2007, Eq. (8)).

In order to proceed, we need a formal definition of a “rescue type”. Analogous to the lemma, we can then derive a recursive formula for the probability that an individual of type (i, j) rescues the process.

#### **Definition 1**

We call an $(i,j)$ individual an $(i,j)$ “rescue type”, denoted as $(i,j,+)$, if

1. it founds an infinite lineage of type $(i,j)$ individuals,
2. there is no individual in its ancestry that founds an infinite lineage of its own type.

We denote by $X_{(i,j,+)}^{(k,l)}$ the number of rescue types $(i,j,+)$ in a process which is founded by an individual of type $(k,l)$.

We define

15 $$\begin{aligned} P_{(i,j)}^{(I,J)}=\text {Prob}(X_{(i,j,+)}^{(I,J)}>0|\text {survival of the process}). \end{aligned}$$

That is, $P_{(i,j)}^{(I,J)}$ gives the probability that there exists an $(i,j)$ rescue type conditioned on survival of the process. A priori, that does not exclude the simultaneous existence of several rescue types. For the following theorem, we again group the recombinant offspring of a type $(I,J)$ individual into two classes: (1) individuals that found a lineage resulting in at least one individual of type $(i,j,+)$ (2) individuals that do not do that. We denote the number of recombinants of the first and second type with $Y_{(i,j,+)}$ and $Y_{(i,j,-)}$, respectively.

#### **Theorem 2**

1. Let $\sigma _{(I,J)}-r_{(I,J)}< 0$. It holds that
  16 $$\begin{aligned} P_{(i,j)}^{(I,J)} = \frac{\left( \sum \nolimits _{k=0}^{I-1}r^{(I,J)}_{(k,J)} (1-Q_{(k,J)})P_{(i,j)}^{(k,J)}+ \sum \nolimits _{k=0}^{J-1} r^{(I,J)}_{(I,k)} (1-Q_{(I,k)}) P_{(i,j)}^{(I,k)}\right) \frac{\frac{\mathrm {d}}{\mathrm {d}s}h(s)|_{s=1}}{r_{(I,J)}} - R_1 }{\left( \sum \nolimits _{k=0}^{I-1}r^{(I,J)}_{(k,J)} (1-Q_{(k,J)})+ \sum \nolimits _{k=0}^{J-1} r^{(I,J)}_{(I,k)} (1-Q_{(I,k)})\right) \frac{\frac{\mathrm {d}}{\mathrm {d}s}h(s)|_{s=1}}{r_{(I,J)}} - R_2 },\nonumber \\ \end{aligned}$$
  where $R_1$ is defined as before and $R_2$ is given by
  17 $$\begin{aligned} R_2 = \frac{\partial }{\partial s_0} \left( \frac{h_2(s_0,s_1)-h_2(0,s_1)}{s_0}\right) \bigg |_{s_0=s_1=1} \end{aligned}$$
  with
  18 $$\begin{aligned} h_2(s_0,s_1) = h(P_{(i,j,+)}s_0 +(1-P_{(i,j,+)})s_1) \end{aligned}$$
  and
  19 $$\begin{aligned} P_{(i,j,+)}=\frac{\sum _{k=0}^{I-1} r_{(k,J)}^{(I,J)} (1-Q_{(k,J)})P_{(i,j)}^{(k,J)} + \sum _{k=0}^{J-1} r_{(I,k)}^{(I,J)} (1-Q_{(I,k)})P_{(i,j)}^{(I,k)}}{r_{(I,J)}}.\nonumber \\ \end{aligned}$$
2. For $\sigma _{(I,J)}-r_{(I,J)}>0$, it holds:
  20 $$\begin{aligned} P_{(I,J)}^{(I,J)} = \frac{1-q_{(I,J)}}{1-Q_{(I,J)}} \end{aligned}$$
  with
  21 $$\begin{aligned} 1-q_{(I,J)}=\frac{\sigma _{(I,J)}-r_{(I,J)}}{1-r_{(I,J)}}, \end{aligned}$$
  where $q_{(I,J)}$ is the unconditioned probability that type $(I,J)$ itself goes extinct. For $(i,j)\ne (I,J)$, it holds:
  22 $$\begin{aligned}&P_{(i,j)}^{(I,J)} = \left( 1-\frac{1-q_{(I,J)}}{1-Q_{(I,J)}}\right) \nonumber \\&\quad \times \frac{\left( \sum \nolimits _{k=0}^{I-1}r^{(I,J)}_{(k,J)} (1-Q_{(k,J)})P_{(i,j)}^{(k,J)}+ \sum \nolimits _{k=0}^{J-1} r^{(I,J)}_{(I,k)} (1-Q_{(I,k)}) P_{(i,j)}^{(I,k)}\right) \frac{\frac{\mathrm {d}}{\mathrm {d}s}\hat{h}(s)|_{s=1}}{r_{(I,J)}} - \hat{R}_1 }{\left( \sum \nolimits _{k=0}^{I-1}r^{(I,J)}_{(k,J)} (1-Q_{(k,J)})+ \sum \nolimits _{k=0}^{J-1} r^{(I,J)}_{(I,k)} (1-Q_{(I,k)})\right) \frac{\frac{\mathrm {d}}{\mathrm {d}s} \hat{h}(s)|_{s=1}}{r_{(I,J)}} - \hat{R}_2 },\nonumber \\ \end{aligned}$$

where $\hat{h}_1$, $\hat{h}_2$, $\hat{R}_1$, and $\hat{R}_2$ are defined analogously to before (using $\hat{h}$ instead of $h$).

#### *Proof*

We first prove part (1) of the theorem. With probability $P_{(i,j,+)}$, a recombinant offspring founds a lineage that generates at least one individual of type $(i,j,+)$. Analogous to before, we obtain

23 $$\begin{aligned} P(Y_{(i,j,+)}>0)&= E[Y_{(i,j,+)}] - R_2\nonumber \\&= P_{(i,j,+)} \frac{\mathrm {d}}{\mathrm {d}s}h(s)|_{s=1}- R_2 \end{aligned}$$

with

24 $$\begin{aligned} R_2=\frac{\partial }{\partial s_0} \left( \frac{h_2(s_0,s_1)-h_2(0,s_1)}{s_0}\right) \bigg |_{s_0=s_1=1}. \end{aligned}$$

It holds:

25 $$\begin{aligned} P^{(I,J)}_{(i,j)} (1-Q_{(I,J)}) = P(Y_{(i,j,+)}>0). \end{aligned}$$

Substituting $1-Q_{(I,J)}$ by the approximation Eq. (7) yields Eq. (16).

If $\sigma _{(I,J)}-r_{(I,J)}>0$, type $(I,J)$ establishes a lineage of its own type with probability

26 $$\begin{aligned} 1-q_{(I,J)}=\frac{\sigma _{(I,J)}-r_{(I,J)}}{1-r_{(I,J)}} \end{aligned}$$

(cf. Lemma 4). It therefore holds:

27 $$\begin{aligned} P_{(I,J)}^{(I,J)} = \frac{1-q_{(I,J)}}{1-Q_{(I,J)}}. \end{aligned}$$

The probability that type $(I,J)$ goes extinct, conditioned on survival of the process, is accordingly given by

28 $$\begin{aligned} P(\text {type (I,J) goes extinct}|\text {survival of the process}) = 1-\frac{1-q_{(I,J)}}{1-Q_{(I,J)}}. \end{aligned}$$

We can now repeat the proof of the first part of the theorem for the process conditioned on extinction of type $(I,J)$. $\square$

#### *Remark 1*

For $\sigma _{(I,J)}-r_{(I,J)}<0$, we have $\frac{\mathrm {d}}{\mathrm {d}s}h(s)|_{s=1}=\frac{r_{(I,J)}}{r_{(I,J)}-\sigma _{(I,J)}}$. For $\sigma _{(I,J)}-r_{(I,J)}>0$, we have $\frac{\mathrm {d}}{\mathrm {d}s}\hat{h}(s)|_{s=1}=\frac{r_{(I,J)}}{\sigma _{(I,J)}-r_{(I,J)}}$.

For $\sigma _{(I,J)}-r_{(I,J)}<0$ not too close to zero, it is likely that only one of the few recombinant offspring of type $(I,J)$ individuals founds an infinite lineage, and we can approximate $P(Y_+\ge 2) \approx 0$ and consequently also $P(Y_{(i,j,+)}\ge 2)\approx 0$. This implies that the error terms $R_1$ and $R_2$ can be ignored. For $\sigma _{(I,J)}-r_{(I,J)}>0$ and $r_{(I,J)}$ small, survival of the process is with high probability contingent on establishment of type $(I,J)$ so that

29 $$\begin{aligned} P_{(i,j)}^{(I,J)} \approx \delta _{I,i}\delta _{J,j} \quad \text {with} \quad \delta _{k_1,k_2} = \left\{ \begin{array}{lll} 1 &{}\quad \text {for}&{} k_1=k_2,\\ 0 &{}\quad \text {else}. \end{array}\right. \end{aligned}$$

We can therefore formulate the following corollary:

#### **Corollary 1**

For $\sigma _{(I,J)}-r_{(I,J)}<0$ not too close to zero and close linkage, we can approximate

30 $$\begin{aligned} P_{(i,j)}^{(I,J)} \approx \frac{P_{(i,j,+)}}{P_\mathrm{success}} = \frac{\sum \nolimits _{k=0}^{I-1}r^{(I,J)}_{(k,J)} (1-Q_{(k,J)})P_{(i,j)}^{(k,J)}+ \sum \nolimits _{k=0}^{J-1} r^{(I,J)}_{(I,k)} (1-Q_{(I,k)}) P_{(i,j)}^{(I,k)} }{\sum \nolimits _{k=0}^{I-1}r^{(I,J)}_{(k,J)} (1-Q_{(k,J)})+ \sum \nolimits _{k=0}^{J-1} r^{(I,J)}_{(I,k)} (1-Q_{(I,k)}) }\nonumber \\ \end{aligned}$$

with

31 $$\begin{aligned} P_{(k,l)}^{(i,j)} \approx \delta _{i,k}\delta _{j,l} \end{aligned}$$

for $\sigma _{(i,j)}-r_{(i,j)}>0$. In this approximation, the $P_{(i,j)}^{(I,J)}$, $i\le I$, $j\le J$, form a probability distribution with

32 $$\begin{aligned} \sum \limits _{i,j} P_{(i,j)}^{(I,J)}=1. \end{aligned}$$

The proof for relation Eq. (32) is given in Appendix C.

The approximation [Eqs. (30) and (31)] implies that exactly one rescue type establishes in the population during the stochastic phase (types with fewer deleterious alleles can still arise later during the deterministic phase), i.e.,

33 $$\begin{aligned} P(X_{(i,j,+)}^{(I,J)}+X_{(k,l,+)}^{(I,J)} > 1|\text {survival}) = 0 \quad \text {for any pair} \quad (i,j), (k,l). \end{aligned}$$

This assumption appears to be justified for a large parameter region with tightly linked deleterious alleles. It is also the basis for most of our analytical treatment of particular cases below. As discussed in Appendix H, the approximation is less accurate if deleterious alleles are relatively loosely linked and/or haplotypes are only slightly deleterious, but in most cases, it introduces only a small error.

We can extend the approximation to include unlinked deleterious alleles and obtain for $\sigma _{(I,J)}-r_{(I,J)}<0$:

34 $$\begin{aligned} P_{(i,j;0)}^{(I,J;F)}&\approx \frac{A+B}{C+D} \nonumber \\ A&= \sum \limits _{l=0}^{F} \left( {\begin{array}{c}F\\ l\end{array}}\right) \left( \frac{1}{2}\right) ^F\!\left\{ \sum \limits _{i=0}^{I-1}r^{(I,J)}_{(i,J)} (1-Q_{(i,J;l)})P_{(i,j;0)}^{(i,J;l)} \right. \nonumber \\&+\left. \sum \limits _{j=0}^{J-1} r^{(I,J)}_{(I,j)} (1-Q_{(I,j;l)})P_{(i,j;0)}^{(I,j;l)}\right\} \nonumber \\ B&= \sum \limits _{l=0}^{F-1}\left( {\begin{array}{c}F\\ l\end{array}}\right) \left( \frac{1}{2}\right) ^F\!(1-r_{(I,J)})(1-Q_{(I,J;l)})P_{(i,j;0)}^{(I,J;l)}\nonumber \\ C&= \sum \limits _{l=0}^{F}\left( {\begin{array}{c}F\\ l\end{array}}\right) \left( \frac{1}{2}\right) ^F\left\{ \sum \limits _{i=0}^{I-1}r^{(I,J)}_{(i,J)} (1-Q_{(i,J;l)}) + \sum \limits _{j=0}^{J-1} r^{(I,J)}_{(I,j)} (1-Q_{(I,j;l)})\right\} \nonumber \\ D&= \sum \limits _{l=0}^{F-1}\left( {\begin{array}{c}F\\ l\end{array}}\right) \left( \frac{1}{2}\right) ^F\!(1-r_{(I,J)})(1-Q_{(I,J;l)}). \end{aligned}$$

For $\sigma _{(I,J)}-r_{(I,J)}>0$, we approximate:

35 $$\begin{aligned} P_{(i,j;0)}^{(I,J;F)}\approx \delta _{I,i}\delta _{J,j}. \end{aligned}$$

### The deterministic phase

It remains to determine whether the haplotype that establishes in the stochastic phase rises to fixation or if types with less deleterious material can establish during the sweep of the beneficial allele. In order to arrive at an approximation for the deterministic phase, we apply and extend an approach developed in Hartfield and Otto (2011). Hartfield and Otto (2011) determined the hitchhiking probability of a single deleterious allele which is closely linked to a beneficial one. For a single hitchhiker, their method can easily be adapted to our model, as shown below. In the Appendix, we further argue that the approach can be extended to a larger number of hitchhikers. Explicit results for two hitchhikers are derived in Appendix F.

For a single potential hitchhiker, assume that type $(0,1)$ with $\sigma _{(0,1)}-r_{(0,1)}>0$ has been introduced and established in the population. Its further growth can be well described deterministically as given by the differential equation Eq. (6). However, in the initial phase, it will on average have grown faster than the deterministic path predicts. Following Uecker and Hermisson (2011), we account for the fast initial increase by the use of an “effective initial population size” $\nu$, which we use as an initial condition for the solution of Eq. (6) (cf. also Desai and Fisher 2007). $\nu$ is an exponentially distributed random variable with

36 $$\begin{aligned} P(\nu \le \nu _0)= 1-\exp {\left( -p_{\text {est}}\nu _0\right) }, \end{aligned}$$

where $p_{\text {est}}=1-q_{(0,1)}$ (cf. Eq. (26)) denotes the establishment probability of a single type $(0,1)$ individual in a wildtype population (Uecker and Hermisson 2011 Eq. (40)). To leading order approximation, we can approximate the distribution by its mean $\bar{\nu }= 1/p_{\text {est}}$. For the relative frequency of type $(0,1)$, it then holds (ignoring recombination):

37 $$\begin{aligned} x_{(0,1)}(t) = \frac{\bar{\nu }\exp {(\sigma _{(0,1)}t)}}{N-\bar{\nu }+\bar{\nu }\exp {(\sigma _{(0,1)}t)}}. \end{aligned}$$

This is a good approximation up to frequency $x_{(0,1)} \approx 1-\frac{\bar{\nu }}{N}$. At higher frequencies, the frequency will again grow faster. Individuals of type $(0,0)$ are recurrently generated by recombination at rate $r_{(0,1)}Nx_{(0,1)}(1-x_{(0,1)})$. As long as they are rare, their dynamics are strongly determined by stochasticity and can once again be approximated by a branching process. Their fitness depends on $x_{(0,1)}(t)$ and hence on time. The dynamics is thus described by a time-inhomogeneous branching process with birth rate $1$ and death rate $1-\sigma _{(0,0)}+x_{(0,1)}(t)\sigma _{(0,1)}$. Following Eq. (16a) in Uecker and Hermisson (2011), the establishment probability of a single individual of type $(0,0)$ generated at time $T$ is given by

38 $$\begin{aligned} p^{(0,0)}_{\text {est}}(T) =\frac{\sigma _{(0,0)}(\sigma _{(0,0)} -\sigma _{(0,1)})}{(\sigma _{(0,0)}-\sigma _{(0,1)})(1-x_{(0,1)}(T)) + \sigma _{(0,0)}x_{(0,1)}(T)}. \end{aligned}$$

“Successful” individuals of type $(0,0)$ are generated at rate

39 $$\begin{aligned} r^{(0,1)}_{(0,0)}Nx_{(0,1)}(t)(1-x_{(0,1)}(t)) p^{(0,0)}_{\text {fix}}(t). \end{aligned}$$

Using this, we obtain for the probability that type $(0,1)$ fixes in the population:

40 $$\begin{aligned} P^{((0,1)\rightarrow (0,1))}_\text {det}&= \exp {\left[ -\int \limits _0^\infty Nr^{(0,1)}_{(0,0)}x_{(0,1)}(t)(1-x_{(0,1)}(t)) p^{(0,0)}_\text {fix}(t)\mathrm {d}t\right] }\nonumber \\&\approx \exp {\left[ -\int \limits _{\bar{\nu }/N}^{1-\bar{\nu }/N} Nr^{(0,1)}_{(0,0)} \frac{1}{\sigma _{(0,1)}}\frac{\sigma _{(0,0)}(\sigma _{(0,0)} -\sigma _{(0,1)})}{ (\sigma _{(0,0)}-\sigma _{(0,1)})(1-x) + \sigma _{(0,0)}x}\mathrm {d}x\right] }\nonumber \\&= \left( \frac{\sigma _{(0,0)}-\frac{\bar{\nu }}{N}\sigma _{(0,1)}}{\sigma _{(0,0)}- \sigma _{(0,1)}(1-\frac{\bar{\nu }}{N})}\right) ^{-\frac{Nr^{(0,1)}_{(0,0)} \sigma _{(0,0)}(\sigma _{(0,0)}-\sigma _{(0,1)})}{\sigma _{(0,1)}^2}}, \end{aligned}$$

where the approximation requires $\bar{\nu }< 0.5 N$. For a single introgressed individual at time $t=0$ in a large population, we can approximate $\bar{\nu }/N \approx 0$ and obtain

41 $$\begin{aligned} P^{((0,1)\rightarrow (0,1))}_\text {det}\approx \left( \frac{\sigma _{(0,0)}}{\sigma _{(0,0)}-\sigma _{(0,1)}}\right) ^{-\frac{Nr^{(0,1)}_{(0,0)} \sigma _{(0,0)}(\sigma _{(0,0)}-\sigma _{(0,1)})}{\sigma _{(0,1)}^2}}. \end{aligned}$$

Eq. (41) corresponds to Eq. (5) in Hartfield and Otto (2011) up to a model-specific factor of $2$ in the exponent if we identify $s_a \equiv \sigma _{(0,0)}$, $s_d \equiv \sigma _{(0,0)}-\sigma _{(0,1)}$, and $r \equiv r_{(0,0)}^{(0,1)}$. If $N(1-q_{(0,1)})\approx N \sigma _{(0,1)}$ is small or if there are already other introgressed haplotypes sweeping in the population as in the generalization to more potential hitchhikers, it makes a quantitative difference whether one accounts for the fast initial increase or not, and we cannot approximate $\bar{\nu }/N \approx 0$ (see Appendix F). Alternatively, one can resort to a diffusion approach for these cases (see Hartfield and Otto 2011 and Appendix G). Note that both approaches assume that recombination is so weak that by itself, it does not influence the frequency path of type $(0,1)$ (i.e., $\sigma _{(0,1)} \gg r_{(0,0)}^{(0,1)}$).

### Concatenation of the stochastic and the deterministic phase

In order to determine which haplotype fixes in the population, we need to concatenate the stochastic and the deterministic phase. Let $\mathcal {A}$ be the set of all types with positive fitness. Type $(k,l)$ establishes in the stochastic phase with probability $P^{(I,J)}_{(k,l)}$ as derived and discussed in Sect. 5.2 and hence enters the deterministic phase. We always assume that only one type does so (and it does so in a single lineage, i.e., there is only one “rescue individual”). Given establishment of type $(k,l)$, we denote by

42 $$\begin{aligned} P_{\text {det}}^{((k,l)\rightarrow (i,j))} \end{aligned}$$

the probability that during the deterministic phase, type $(i,j)\in \mathcal {A}$ is generated and finally fixes in the population. Summing over all $(k,l)\in \mathcal {A}$ yields:

43 $$\begin{aligned} P(\text {``type (i,j) fixes''}) = \sum \limits _{(k,l)\in \mathcal {A}} P_{(k,l)}^{(I,J)} P_{\text {det}}^{((k,l) \rightarrow (i,j))}. \end{aligned}$$

If not stated otherwise, the results presented in Sect. 6 are based on Eq. (43) with $P_{(k,l)}^{(I,J)}$ obtained by Eqs. (30) and (31). The recursions are performed by a program written in the C programming language. Approximations for $P_{\text {det}}^{((0,2)\rightarrow (0,\cdot ))}$ with $\sigma _{(2,0)}-r_{(0,2)}>0$ and $P_{\text {det}}^{((1,1)\rightarrow (\cdot ,\cdot ))}$ with $\sigma _{(1,1)}-r_{(1,1)}>0$ are derived in Appendix F. All numerical evaluation of the integrals that appear in these approximations is done in Mathematica (Wolfram Research, Champaign, USA). The accuracy of the approach and the appropriateness of the assumptions are addressed in Appendix H.

## Application to various biological scenarios

### The impact of unlinked alleles

If $I=J=0$, the extinction probability is given by Eq. (5). For $Q_0$, $Q_1$ and $Q_2$, we obtain:

44a $$\begin{aligned} Q_0&= 1-\sigma _0, \end{aligned}$$

44b $$\begin{aligned} Q_1&= 2-\sigma _1-\frac{1}{2}Q_0 - \sqrt{\left( 2-\sigma _1-\frac{1}{2}Q_0\right) ^2-2(1-\sigma _1)}, \end{aligned}$$

44c $$\begin{aligned} Q_2&= 4-2\sigma _2-\frac{1}{2}Q_0-Q_1-2\sqrt{\left( \frac{1}{4}Q_0+\frac{1}{2}Q_{1}-2+\sigma _2\right) ^2-(1-\sigma _2)}. \qquad \end{aligned}$$

**Fig. 2:**
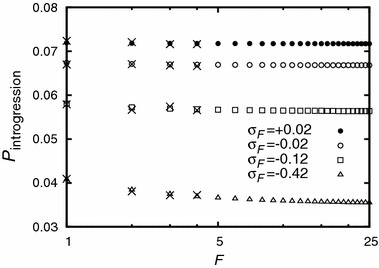
The introgression probability as a function of the number of unlinked deleterious alleles. The total effect on fitness is kept constant; $\sigma _F$ denotes the Malthusian fitness of a haplotype carrying the adaptive allele and $F$ unlinked deleterious alleles. The introgression probability is approximately the same whether the effect is distributed over few strongly or many slightly deleterious alleles. The advantageous allele has Malthusian fitness $\sigma _0=0.08$. In the absence of deleterious alleles, it would establish with probability $1-Q_0 = \sigma _{(0,0)}=0.08$. The *crosses* denote simulation results. Each simulation point is the average of $10^6$ introgression attempts

How does the number of unlinked deleterious alleles impact the introgression probability if their total effect is kept constant? A comparison of $1-Q_{1}$ with $\sigma _1=\sigma _0-2s_{\text {del}}$ and $1-Q_{2}$ with $\sigma _1=\sigma _0-s_{\text {del}}$ and $\sigma _2=\sigma _0-2s_{\text {del}}$ yields:

45 $$\begin{aligned} (1-Q_2(s_{\text {del}}))/(1-Q_1(2s_{\text {del}}))=1+\mathcal {O}(s_{\text {del}}^2), \end{aligned}$$

i.e., unless the deleterious effect is very strong, the establishment probability is approximately the same for both scenarios (either one deleterious allele of effect $2s_{\text {del}}$ or two deleterious alleles each of effect $s_{\text {del}}$). Figure 2 generalizes this result to $F>2$. One sees that unlinked alleles significantly reduce the introgression probability. However, it is irrelevant whether there is one strongly deleterious allele or many slightly deleterious alleles. By how much do unlinked deleterious alleles of compound effect $S_{\text {del}}$ reduce the introgression probability? Making use of the previous observation, it is sufficient to consider a single unlinked allele of effect $S_{\text {del}}$. A Taylor expansion yields:

46 $$\begin{aligned} \frac{1-Q_1}{1-Q_0} = 1-\frac{2}{1+\sigma _0}S_{\text {del}} + \mathcal {O}(S_{\text {del}}^2) \approx 1- 2 S_{\text {del}} + \mathcal {O}(S_{\text {del}}^2), \end{aligned}$$

i.e., unlinked alleles approximately reduce the introgression probability by a factor that is independent of $\sigma _0$.

**Fig. 3:**
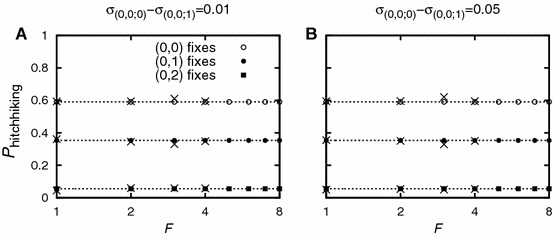
The hitchhiking probability as a function of the number of unlinked deleterious alleles. The *dotted lines* correspond to the respective values for $F=0$. Each unlinked deleterious allele reduces fitness by $-0.01$ (Panel **a**) and $-0.05$ (Panel **b**), respectively. Unlinked alleles do not visibly impact the hitchhiking probability of closely linked deleterious alleles. Parameter values are: $I=0$, $J=3$, $\sigma _0=0.075$, $\sigma _{(0,1)}=0.07$, $\sigma _{(0,2)}=0.05$, $\sigma _{(0,3)}=-0.015$, $N=$10,000, $r_{(\cdot ,\cdot )}^{(\cdot ,\cdot )}=0.0001$. The *crosses* denote simulation results. Each simulation point is the average of 2,000 successful introgression events

While unlinked alleles have a significant impact on the probability of adaptive gene introgression ($\sim$10–50 % in Fig. 2), they do not visibly influence the hitchhiking probability of closely linked deleterious alleles (cf. Fig. 3).

### The impact of a single linked deleterious allele

In this section, we consider the impact of a single linked deleterious allele (cf. also Iwasa et al. 2004a). From Eq. (4), we obtain:

47a $$\begin{aligned} Q_{(0,0)}&= 1-\sigma _{(0,0)}, \end{aligned}$$

47b $$\begin{aligned} Q_{(0,1)}&= \frac{1}{2(1-r_{(0,1)})} \bigg ( 2-\sigma _{(0,1)}-r_{(0,1)}Q_{(0,0)} \end{aligned}$$

47c $$\begin{aligned}&-\sqrt{\left( 2-\sigma _{(0,1)}-r_{(0,1)}Q_{(0,0)}\right) ^2-4(1-\sigma _{(0,1)})(1-r_{(0,1)})} \bigg ) \end{aligned}$$

47d $$\begin{aligned}&\approx \left\{ \begin{array}{lll} (1-\sigma _{(0,1)})\left( 1-\frac{\sigma _{(0,0)}-\sigma _{(0,1)}}{\sigma _{(0,1)}}r_{(0,1)}\right) &{} \text {for} &{}\sigma _{(0,1)}>0,\\ 1+\frac{\sigma _{(0,0)}}{\sigma _{(0,1)}}r_{(0,1)} &{} \text {for} &{} \sigma _{(0,1)}<0. \end{array}\right. \end{aligned}$$

The approximation is a first order Taylor expansion in $r_{(0,1)}$, which yields accurate results for small $r_{(0,1)}$ if $\sigma _{(0,1)}$ is not too close to zero. Due to the assumption of single crossover only, $Q_{(0,1)}$ exactly corresponds to $Q_{1}$ for $r_{(0,1)}=0.5$ (where $\sigma _{(0,0)} \equiv \sigma _{0}$ and $\sigma _{(0,1)}\equiv \sigma _1$). How does a single deleterious allele impact the probability of adaptive gene introgression? We can measure the impact by the relative reduction of the introgression probability

48 $$\begin{aligned} \Delta P = 1 - \frac{1-Q_{(0,1)}}{1-Q_{(0,0)}} = 1 - \frac{1-Q_{(0,1)}}{\sigma _{(0,0)}}. \end{aligned}$$

If $\Delta P$ is close to zero, the deleterious allele has a weak impact; if $\Delta P$ is close to one, it has a strong impact. The influence is obviously strongest for $r_{(0,1)}=0$. If $\sigma _{(0,1)}>0$, the maximum relative reduction in the introgression probability is given by $s_{\text {del}}/\sigma _{(0,0)}$ with $s_{\text {del}}:=\sigma _{(0,0)}-\sigma _{(0,1)}$. For $\sigma _{(0,1)}\le 0$ and $r_{(0,1)}=0$, the advantageous allele can not introgress at all (except through fixation by drift which is not considered here). For tight linkage, we use the foregoing Taylor expansion and obtain

49a $$\begin{aligned} \Delta P&\approx \frac{s_{\text {del}}}{\sigma _{(0,0)}} \left( 1-\frac{r_{(0,1)}}{\sigma _{(0,1)}} \right) \qquad \text {for} \quad \sigma _{(0,1)}>0, \end{aligned}$$

49b $$\begin{aligned} \Delta P&\approx 1 + \frac{r_{(0,1)}}{\sigma _{(0,1)}}\qquad \qquad \qquad \quad \, \text {for} \quad \sigma _{(0,1)}<0. \end{aligned}$$

The maximum impact is strongest for a weak beneficial mutation (where introgression is easily reduced to zero for tight linkage). The impact gets weaker with increasing recombination on the scale of $\sigma _{(0,1)}$. I.e., if either $\sigma _{(0,0)} \gg s_{\text {del}}$, or if $s_{\text {del}} \gg \sigma _{(0,0)}$, the impact declines only slowly. In order to determine the behavior for strong recombination, we perform a Taylor expansion of $\Delta P$ in $s_{\text {del}}$:

50 $$\begin{aligned} \Delta P = \frac{1}{r_{(0,1)}+ \sigma _{(0,0)}(1-r_{(0,1)})}s_{\text {del}} + \mathcal {O}\left( s_{\text {del}}^2\right) . \end{aligned}$$

For $r_{(0,1)} \gg \sigma _{(0,0)}$, the relative reduction becomes independent of the strength of the beneficial allele. This is in strong contrast to the behavior for the tight linkage case. The impact declines on a scale of $s_{\text {del}}$. It becomes irrelevant if $r_{(0,1)} \gg s_{\text {del}}$. An unlinked deleterious allele leads to a relative reduction of $2s_{\text {del}}$, which can still be appreciable if the deleterious mutation has a strong effect.

If $\sigma _{(0,1)}-r_{(0,1)}>0$, the deleterious allele can hitchhike to fixation. For the assessment of its hitchhiking probability, we do not follow the simple approximation (31) but give a detailed analysis of the stochastic establishment phase instead. Type $(0,1)$ establishes with probability

51 $$\begin{aligned} 1-q_{(0,1)} = \frac{\sigma _{(0,1)}-r_{(0,1)}}{1-r_{(0,1)}}, \end{aligned}$$

i.e.,

52 $$\begin{aligned} P^{(0,1)}_{(0,1)} = \frac{\sigma _{(0,1)}-r_{(0,1)}}{1-r_{(0,1)}} \frac{1}{1-Q_{(0,1)}}, \end{aligned}$$

and

53 $$\begin{aligned} P_{\text {hitchhiking}} = P^{(0,1)}_{(0,1)} P_{\text {det}}^{((0,1)\rightarrow (0,1))}. \end{aligned}$$

In order to asses the relevance of the stochastic and the deterministic phases, we perform a first-order Taylor expansion of $P_{(0,1)}^{(0,1)}$ in $r_{(0,1)}$:

54 $$\begin{aligned} P_{(0,1)}^{(0,1)} \approx 1- r_{(0,1)} \sigma _{(0,0)}\frac{1-\sigma _{(0,1)}}{\sigma _{(0,1)}^2} \approx 1- r_{(0,1)}\frac{\sigma _{(0,0)}}{\sigma _{(0,1)}^2}. \end{aligned}$$

**Fig. 4:**
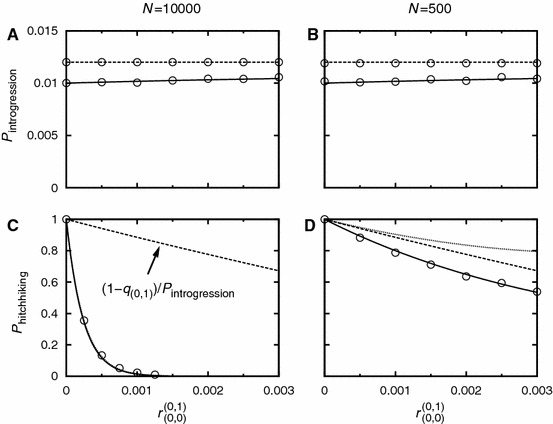
Panels **a** and **b**: The introgression probability as a function of linkage. For the *solid line*: $I=0, J=1$. For the *dashed line*: $I=J=0$. Panels **c** and **d**: The hitchhiking probability of a single deleterious allele ($I=0$, $J=1$). The *solid line* represents the hitchhiking probability as given by Eq. (53). The *dashed* and the *dotted lines* show the impact of the stochastic phase [Eq. (52)] and the deterministic phase [Eq. (40)], respectively. In Panel **c**, the *solid* and the *dotted line* are virtually indistinguishable; the deterministic phase dominates. In Panel **d**, the stochastic phase has a significant impact on the hitchhiking probability. Parameter values are: $N=10,000$ (Panels **a** and **c**), $N=500$ (Panels **b** and **d**), $\sigma _{(0,0)}=0.012$, $\sigma _{(0,1)}=0.01$. The *circles* denote simulation results. For Panels **a** and **b**, each simulation point is the average of $10^6$ introgression attempts. For Panels **c** and **d**, each simulation point is the average of $10^4$ successful introgression events

I.e., changes in $P_{(0,1)}^{(0,1)}$ occur on the scale of $r_{(0,1)}\sim \sigma _{(0,1)}^2/\sigma _{(0,0)}$. For the deterministic phase [Eq. (41)], the scale is set by $r_{(0,1)} \sim \sigma _{(0,1)}^2/(N\sigma _{(0,0)}s_{\text {del}})$. This allows us to distinguish two parameter regimes: if $N s_{\text {del}} \gg 1$, the deterministic phase dominates. However, if $N s_{\text {del}} \approx 1$ or smaller, the stochastic phase cannot be ignored. Figures 4C and D illustrate how the stochastic and deterministic phases combine to form the probability of hitchhiking for $N=10000$ and $N=500$, respectively. For $N=$10,000, the behavior is dominated by the deterministic phase which decays quickly as a function of $r_{(0,1)}$. In the parameter range where $P_{\text {det}}^{((0,1)\rightarrow (0,1))}$ is appreciable, one can ignore the influence of the stochastic phase. For $N=500$, however, $P_{\text {det}}^{((0,1)\rightarrow (0,1))}$ decays slowly, and the stochastic phase has a non-negligible impact on hitchhiking: e.g., for $r_{(0,1)}=0.003$, we find $P_{\text {det}}^{((0,1)\rightarrow (0,1))} \approx 0.8$ and $P^{(0,1)}_{(0,1)} P_{\text {det}}^{((0,1)\rightarrow (0,1))} \approx 0.53$. Note also that if $N(1-q_{(0,1)})$ is small, we have to account for deviations from the deterministic path. These deviations can be accounted for via the parameter $\bar{\nu }$ as in Eq. (40) or via a diffusion approach as in Hartfield and Otto (2011). In Appendix G, we give the diffusion equation adjusted to our model and compare the result to Eq. (40).

A comparison between Panels A/B with Panels C/D of Fig. 4 shows that the introgression probability changes only slightly over the depicted range of recombination, while the hitchhiking probability significantly decreases with increasing recombination distance; the scale is strongly affected by the population size.

### The impact of a second deleterious allele

**Fig. 5:**
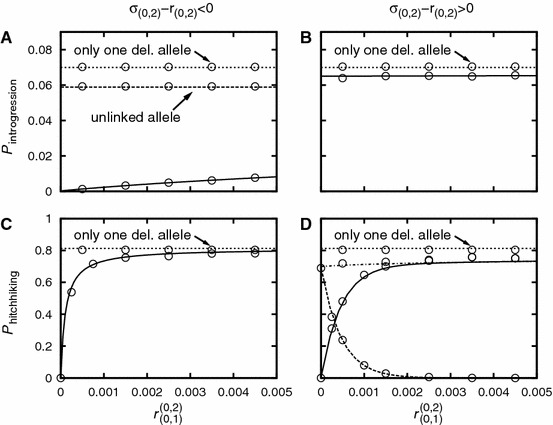
Panels **a** and **b**: The introgression probability of an adaptive allele linked to two deleterious alleles. Panels **c** and **d**: The hitchhiking probability of a closely linked deleterious allele. In each Panel, the *short-dashed line* gives the respective probability in the absence of the second deleterious allele. In Panel **a**, the *long-dashed line* gives the introgression probability for one closely linked and one unlinked allele (in Panel **b**, this line would be indistinguishable from the *short-dashed line*). In Panel **d**, the *solid line* gives the probability that the haplotype $(0,1)$ fixes. The *long-dashed line* gives the probability that the haplotype $(0,2)$ fixes. The *dash-dotted line* gives the probability that either of the two fixes. For Panels **a** and **c**: $\sigma _{(0,2)}=-0.03$. For Panels **b** and **d**: $\sigma _{(0,2)}=0.065$. The other parameter values are: $\sigma _{(0,0)}=0.075$, $\sigma _{(0,1)}=0.07$, $N=$10,000, $r_{(0,0)}^{(0,1)}=0.0001$. The *circles* denote simulation results. For Panels **a** and **b**, each simulation point it the average of $10^6$ introgression attempts. For Panels, **c** and **d**, each simulation point is the average of 2,000 successful introgression events

**Fig. 6:**
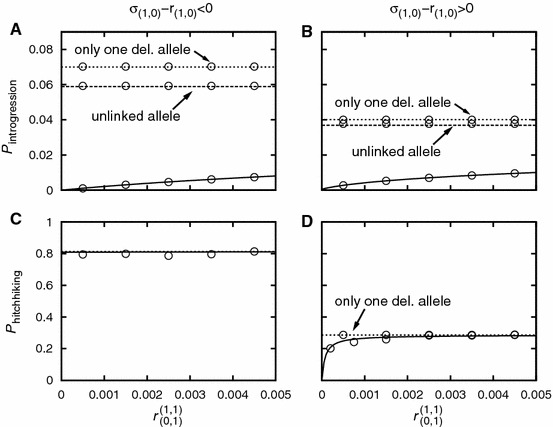
Panels **a** and **b**: The introgression probability of an adaptive allele linked to two deleterious alleles. Panels **c** and **d**: The fixation probability of type $(0,1)$. In each Panel, the *short-dashed line* gives the respective probability in the absence of the second deleterious allele ($I=0$). In Panels **a** and **b**, the *long-dashed line* gives the introgression probability for one closely linked and one unlinked allele. For Panels **a** and **c**: $\sigma _{(0,1)}=0.07$, $\sigma _{(1,0)}=-0.025$, $\sigma _{(1,1)}=-0.03$, i.e., type $(1,0)$ cannot rise to fixation. For Panels **b** and **d**: $\sigma _{(0,1)}=0.04$, $\sigma _{(1,0)}=0.03$, $\sigma _{(1,1)}=-0.005$, i.e., either deleterious allele can fix but not both. The other parameter values are: $\sigma _{(0,0)}=0.075$, $N=$10,000, $r_{(1,0)}^{(1,1)}=0.0001$. The *circles* denote simulation results. For Panels **a** and **b**, each simulation point it the average of $10^6$ introgression attempts. For Panels, **c** and **d**, each simulation point is the average of 2,000 successful introgression events

**Fig. 7:**
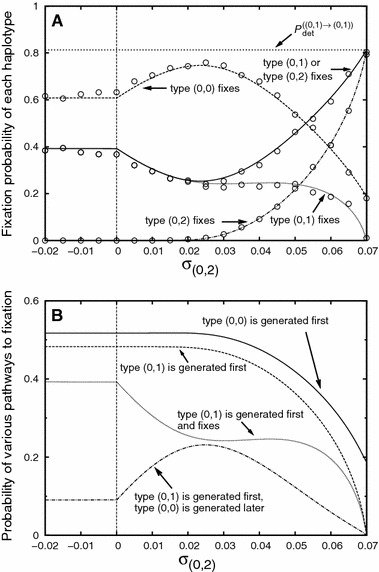
Panel **a**: The hitchhiking probability of closely linked deleterious alleles as a function of the Malthusian fitness parameter $\sigma _{(0,2)}$. The *plot* shows the probabilities with which the various haplotypes fix in the population. Panel **b**: An illustration of the respective pathways of recombination and establishment that lead to fixation of types $(0,0)$ and $(0,1)$: by recombination, either a successful lineage of type $(0,0)$ (*solid line*) or type $(0,1)$ (*dashed line*) can be generated. In the first case, fixation of type $(0,0)$ is certain. In the second case, a successful lineage of type $(0,0)$ can still be generated later and outcompete type $(0,1)$ (*dashed-dotted line*). This scenario is particularly likely if type $(0,2)$ has an intermediate fitness (see text). The *dotted curves* in Panel **a** and **b** show the same probability. Parameter values are: $I=0$, $J=2$, $\sigma _{(0,0)}=0.075$, $\sigma _{(0,1)}=0.07$, $N=$10,000, $r_{(0,0)}^{(0,1)}=r^{(0,2)}_{(0,1)}=0.0001$. The *circles* denote simulation results. Each simulation point is the average of 2,000 successful introgression events

**Fig. 8:**
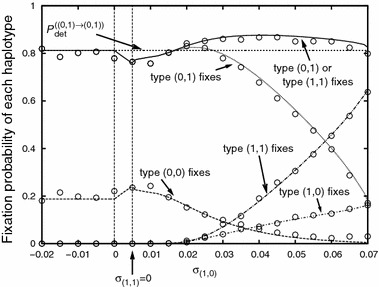
The hitchhiking probability of closely linked deleterious alleles as a function of the Malthusian fitness parameter $\sigma _{(1,0)}$. The plot shows the probabilities with which the various haplotypes fix in the population. Parameter values are: $I=1$, $J=1$, $\sigma _{(0,0)}=0.075$, $\sigma _{(0,1)}=0.07$, $N=$10,000, $r_{(0,0)}^{(0,1)}=r^{(1,0)}_{(0,0)}=0.0001$. The *circles* denote simulation results. Each simulation point is the average of 2,000 successful introgression events

In this section, we investigate how a second deleterious allele affects the introgression and the hitchhiking probability in dependence of the strength of selection, the genetic architecture, and linkage. The results are summarized in Figs. 5, 6, 7, 8.

Figures 5 and 6 consider the dependence on linkage. One sees that a strongly deleterious allele significantly affects the introgression probability even if it is only loosely linked. The impact on the hitchhiking probability is more subtle, and several cases must be distinguished. We first turn to Fig. 5, in which the second deleterious allele is on the same side of the beneficial allele as the first one. Panel C shows the behavior for $\sigma _{(0,2)}-r_{(0,2)}<0$. In that case, applying Eq. (30) with Eq. (31), it approximately holds:

55 $$\begin{aligned} P_{(0,1)}^{(0,2)} \!\approx \! \frac{r_{(0,1)}^{(0,2)}(1-Q_{(0,1)})}{ r_{(0,0)}^{(0,2)}(1-Q_{(0,0)}) + r_{(0,1)}^{(0,2)}(1-Q_{(0,1)})} \!\approx \! \frac{r_{(0,1)}^{(0,2)}\sigma _{(0,1)}}{r_{(0,0)}^{(0,2)}\sigma _{(0,0)} + r_{(0,1)}^{(0,2)}\sigma _{(0,1)}},\qquad \end{aligned}$$

where we have used $Q_{(0,1)}\approx 1-\sigma _{(0,1)}$. Hence:

56 $$\begin{aligned} P_{\text {hitchhiking}}^{(0,2)}&\approx \frac{r_{(0,1)}^{(0,2)}\sigma _{(0,1)}}{r_{(0,0)}^{(0,2)}\sigma _{(0,0)} + r_{(0,1)}^{(0,2)}\sigma _{(0,1)}} P_{\text {det}}^{((0,1)\rightarrow (0,1))}\nonumber \\&\approx \frac{r_{(0,1)}^{(0,2)}\sigma _{(0,1)}}{r_{(0,0)}^{(0,2)}\sigma _{(0,0)} + r_{(0,1)}^{(0,2)}\sigma _{(0,1)}} P_{\text {hitchhiking}}^{(0,1)}, \end{aligned}$$

where $P_{\text {hitchhiking}}^{(\cdot ,\cdot )}$ denotes the probability that type $(0,1)$ fixes in the population given that type $(\cdot ,\cdot )$ got initially introduced. I.e., for close linkage of the second deleterious allele, the hitchhiking probability gets strongly reduced. However, as linkage gets looser, it converges quickly to its value in the absence of the second allele, $P_{\text {det}}^{((0,1)\rightarrow (0,1))}$. Let $c$ denote the factor by which the second deleterious allele reduces the hitchhiking probability of the first one:

57 $$\begin{aligned} c = \frac{P_{\text {hitchhiking}}^{(0,1)}-P_{\text {hitchhiking}}^{(0,2)} }{P_{\text {hitchhiking}}^{(0,1)}}. \end{aligned}$$

By rearranging terms, we obtain

58 $$\begin{aligned} \frac{r_{(0,1)}^{(0,2)}}{r_{(0,0)}^{(0,2)}} = \frac{1-c}{c} \frac{\sigma _{(0,0)}}{\sigma _{(0,1)}} \quad \Leftrightarrow \quad c =\frac{1}{1+\frac{r_{(0,1)}^{(0,2)}\sigma _{(0,1)}}{r_{(0,0)}^{(0,2)}\sigma _{(0,0)}}}. \end{aligned}$$

Importantly, the strength of selection of the second deleterious allele has no effect (as long as it is strong enough for our approximation to apply), and the other selection coefficients play only a minor role. The second deleterious allele is relevant if

59 $$\begin{aligned} r_{(0,1)}^{(0,2)} < r_{(0,0)}^{(0,1)} \frac{\sigma _{(0,0)}}{\sigma _{(0,1)}} \approx r_{(0,0)}^{(0,1)} \quad \text {for} \quad \sigma _{(0,0)} \gg s_{\text {del}}, \end{aligned}$$

which is independent of the selection coefficients if $\sigma _{(0,0)} \gg s_{\text {del}}$. The hitchhiking probability is crucially determined by the ratio of recombination distances $r_{(0,1)}^{(0,2)}/r_{(0,0)}^{(0,1)}$.

In Panel D, the second deleterious allele can hitchhike to fixation, too ($\sigma _{(0,2)}-r_{(0,2)}>0$). The total hitchhiking probability of the closest deleterious alleles (i.e., the probability that either type $(0,1)$ or type $(0,2)$ fixes) is then only moderately reduced. For $r_{(0,1)}^{(0,2)}$ small, both alleles fix. With increasing recombination distance the analytical result underestimates the true hitchhiking probability (see Appendix H).

In Fig. 6, the beneficial allele is flanked by the two deleterious alleles. If one of the alleles is strongly deleterious, the hitchhiking probability of the other one is not visibly reduced (Panel C). This is because for successful introgression, the strongly deleterious allele has to recombine away very early. The situation in Panel D ($\sigma _{(0,1)}-r_{(0,1)}>0$, $\sigma _{(1,0)} -r_{(1,0)}>0$, but $\sigma _{(1,1)}-r_{(1,1)}<0$) looks similar to Fig. 5C. We have

60 $$\begin{aligned} P_{\text {hitchhiking}}^{(1,1)} \approx \frac{r_{(0,1)}^{(1,1)}\sigma _{(0,1)}}{r_{(1,0)}^{(1,1)}\sigma _{(1,0)} + r_{(0,1)}^{(1,1)}\sigma _{(0,1)}} P_{\text {hitchhiking}}^{(0,1)}. \end{aligned}$$

This is formally similar to Eq. (56). There is, however, an important difference: Now, the selection coefficient of the second deleterious allele is crucial. Its influences ceases with increasing strength as $\sigma _{(1,0)}$ goes to zero. If both deleterious alleles have approximately the same effect ($\sigma _{(0,1)} \approx \sigma _{(1,0)}$), the behavior is again determined by the ratio of the recombination distances $r_{(0,1)}^{(1,1)}/r_{(1,0)}^{(1,1)}$.

Figures 7 and 8 show how the selective disadvantage of a second closely linked deleterious allele affects the hitchhiking probability. If it is on the same side of the beneficial allele as the first one (Fig. 7), the hitchhiking probability of the first one is greatly reduced unless the selective disadvantage is very slight ($\sigma _{(0,2)}\approx \sigma _{(0,1)}$). The reduction is greatest for intermediate values of the selection coefficient (see Fig. 7a). In this parameter regime, type $(0,2)$ significantly increases in frequency before a successful lineage of type $(0,0)$ or $(0,1)$ is generated. As a consequence, the time to fixation of the beneficial allele is relatively long. Even if a successful lineage of type $(0,1)$ can establish, it is therefore likely that later, a successful lineage of type $(0,0)$ is generated (see Fig. 7b for an illustration of this reasoning). If the beneficial allele is flanked at equal small recombination distances by two deleterious alleles as in Fig. 8, the total hitchhiking probability of the deleterious allele to the right (fixation of type $(0,1)$ or type $(1,1)$) is barely influenced by the presence of the second deleterious allele, irrespective of the selective disadvantage of the latter. Note, however, that recombination is weak in Fig. 8. For strong recombination and $\sigma _{(0,1)}\approx \sigma _{(1,0)}$, it is not unlikely that both a successful lineage of type $(0,1)$ and of type $(1,0)$ establish and coexist for a long time, making the production of a successful $(0,0)$ recombinant very likely (see Fig. 13 in Appendix H).

### The impact of several linked deleterious alleles

To start with, assume $\sigma _{(I,J)}-r_{(I,J)}>0$. If all recombination distances are small, we can generalize the result Eq. (41) and calculate the probability that all deleterious alleles hitchhike to fixation. Analogous to the derivation of Eq. (41), we obtain for the probability that all deleterious alleles hitchhike to fixation

61 $$\begin{aligned} Q_{\text {det}}^{(I,J)}&= \prod \limits _{i=0}^{I-1} \left( \frac{\sigma _{(i,J)}}{\sigma _{(i,J)}-\sigma _{(I,J)}} \right) ^{-\frac{Nr_{(i,J)}^{(I,J)}\sigma _{(i,J)}(\sigma _{(i,J)}-\sigma _{(I,J)})}{\sigma _{(I,J)}^2}}\!\! \nonumber \\&\times \prod \limits _{j=0}^{J-1} \left( \frac{\sigma _{(I,j)}}{\sigma _{(I,j)}-\sigma _{(I,J)}} \right) ^{-\frac{Nr_{(I,j)}^{(I,J)}\sigma _{(I,j)}(\sigma _{(I,j)}-\sigma _{(I,J)})}{\sigma _{(I,J)}^2}}. \end{aligned}$$

We now turn to $\sigma _{(I,J)}-r_{(I,J)}<0$ and study several selected scenarios which impart a general intuition. Consider first the special case $I=0$, $\sigma _{(0,j)}-r_{(0,j)} >0$, and $\sigma _{(0,l)}-r_{(0,l)}<0$, $l>j$, i.e., all deleterious alleles are located at one side of the adaptive allele and at most $j$ of them can potentially hitchhike to fixation. For tight linkage, we can again approximate $1-Q_{(0,k)}\approx \sigma _{(0,k)}$ for $k\le j$ and obtain (proof by induction):

62 $$\begin{aligned} P_{(0,k)}^{(0,J)} \approx \frac{r_{(0,k)}^{(0,J)}\sigma _{(0,k)}}{\sum \limits _{l=0}^{j} r_{(0,l)}^{(0,J)}\sigma _{(0,l)}}. \end{aligned}$$

I.e., the selection coefficients of the $(j+1)$th, $(j+2)$th, ..., $J$th alleles do not enter the result. Furthermore, it does not matter where the deleterious alleles beyond the $(j+1)$th are located.

As another example, consider the special case $J=1$, $\sigma _{(0,1)}-r_{(0,1)} >0$, and $\sigma _{(i,j)}-r_{(i,j)}<0$, $i>0$. If linkage is tight, the hitchhiking probability is barely reduced by additional deleterious mutations.

**Fig. 9:**
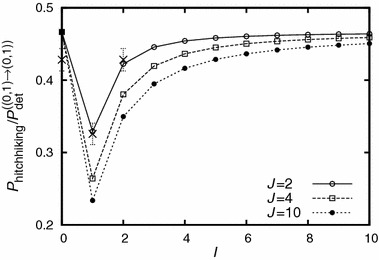
The reduction of the hitchhiking probability of a closely linked deleterious allele due to the impact of other deleterious alleles. On the $x$ axis, the number $I$ of deleterious alleles to the left of the beneficial allele is varied. Only types $(0,0)$ and $(0,1)$ have positive fitness. The fixation probability of type $(0,1)$ conditioned on fixation of the adaptative allele ($P_{\text {hitchhiking}}$) displays a pronounced dip for $I=1$. Parameter values are: $\sigma _{(0,0)}=0.08$, $\sigma _{(0,1)}=0.07$, $\sigma _{(0,2)}=-0.005$, $\sigma _{(1,0)}=-0.002$, all other deleterious alleles have an effect of $-0.01$, $r_{(i,j)}^{(k,l)}=0.0001$ for all $k,l,i,j$, $N=$10,000. The *crosses* denote simulation results. Each simulation point is the average of 1,000 successful introgression events. The introgression probability for the scenarios shown in the plot ranges from $\approx 1.3\cdot 10^{-7}$ ($I=J=10$) to $\approx 0.002$ ($I=0$, $J=2$), i.e., 1,000 successful introgression events correspond to $\approx 500,000$ to $6 \cdot 10^9$ introgression attempts; simulations for complex scenarios are hence impractical

Finally, let $\sigma _{(0,1)}-r_{(0,1)} >0$, $I$ and $J\ge 1$ arbitrary, and $\sigma _{(i,j)}-r_{(i,j)}<0$ for $(i,j)\notin \left\{ (0,0),(0,1)\right\}$. Figure 9 shows how additional deleterious alleles that can themselves not hitchhike to fixation can influence the hitchhiking probability of a slightly deleterious allele. The pattern can be understood by consideration of the various paths which lead to establishment of the beneficial allele: unless $I=0$ (or $J=1$), at least two recombination events are necessary to generate a type with positive Malthusian fitness. The position of the first successful recombination event depends on the fitness of the types that are generated by recombination. Since $\sigma _{(1,0)}$ is only slightly deleterious, the first recombination event is likely to generate this type if $I=1$. In this case, the hitchhiking probability is strongly reduced (cf. the dip in Fig. 9). For $I>1$, however, type $(1,0)$ cannot be generated via a single recombination event, and the reduction is less pronounced, getting smaller with increasing $I$. For large $I$, adding more deleterious alleles to the left or the right has only a weak effect. Generally, deleterious alleles that render haplotypes strongly disfavored have to be lost as quickly as possible, and the pathway to establishment of the beneficial allele does usually not involve tunneling via more strongly deleterious haplotypes than necessary. At either side of the adaptive allele, consider the set of alleles which must be lost for establishment. As a rule of thumb, each of the two sets can be replaced by a “virtual” allele of the respective compound effect. In order to have the same effect as the set of actual alleles, this virtual allele has to be located at the same position as the deleterious allele (out of the set) which is closest to the adaptation.

## Discussion

Gene flow between related species is frequent in nature. Although many foreign alleles burden their carrier with a selective disadvantage, exchange of genetic material between populations is often still possible. If neutral or advantageous alleles survive the fitness bottleneck caused by linked or unlinked deleterious alleles they can become permanently incorporated into the genome of the sister species. Picking up locally adaptive alleles from an indigenous species can help species to expand their range to previously uninhabitable regions (e.g., Heiser 1951; Whitney et al. 2006). Adaptive gene introgression is hence a clever evolutionary mechanism that can speed up adaptation to novel environments. Human activities create ample opportunity for hybridization between domestic animals or crop plants with their wild relatives (e.g. Fitzpatrick et al. 2010; Ellstrand et al. 1999, 2013). In this context, the introgression of alleles from genetically modified organisms (e.g., insecticide resistance genes) into weedy species is recognized as a risk that can cause permanent ecological damage. A quantitative analysis of the introgression process is essential both to assess the importance of adaptive gene introgression as an evolutionary pathway to adaptation and to estimate the ecological risks associated with unwanted hybridization.

The flow of adaptive alleles among species is hampered by the reduced fitness of inter-species crosses. This reduction in fitness is due to alleles from the donor species that are deleterious in the new environment (which we consider in this paper) and/or the new genomic background. If their compound effect outweighs the benefits of the adaptation, some deleterious alleles must be eliminated by recombination in hybrid back-crosses before the favorable allele can establish. However, closely linked slightly deleterious alleles might be dragged along to fixation. In this paper, we developed a framework to investigate the role of linked and unlinked deleterious alleles in adaptive gene introgression. The model accounts for an explicit genetic structure and describes the genetic evolution of a haploid population under the influence of selection, recombination, and drift after a single hybridization event. The analysis is based on the theory of branching processes. The early phase of spread of the advantageous allele is approximated by a reducible multitype branching process with a special structure: within the branching process approximation, offspring are either of the same type as their parent or carry fewer deleterious alleles (for similar setups see Barton and Bengtsson 1986; Demon et al. 2007; Gosh and Haccou 2010; Gosh et al. 2012a, b; Yanchukov and Proulx 2014). The fate of the recombinants that are generated after the initial establishment and until fixation of the beneficial allele is modeled by a time-inhomogeneous single-type branching process. For the analysis of the first phase, we make use of methods developed by Serra (2006) and Serra and Haccou (2007). The analysis of the second phase builds on work by Hartfield and Otto (2011). The combination of the results from both phases allows for the analytical treatment of the entire process.

*The introgression probability* How likely is it that the advantageous allele can establish itself in the population? For large populations, the probability of adaptive gene introgression only depends on the early phase of the spread, where the dynamics are well described by a branching process. Technically, this is similar to studies on scenarios where adaptation relies on the accumulation of new mutations via stochastic tunneling. The gain of adaptive alleles by mutation in these scenarios, which include the crossing of fitness valleys (e.g., Weissman et al. 2009; Proulx 2011), tumor initiation (e.g., Iwasa et al. 2003, 2004a, b), or adaptation of a pathogen to a new host (e.g., Antia et al. 2003), corresponds to the removal of deleterious alleles by recombination in models of gene introgression. The survival probability of a multitype branching process is in general difficult to determine, and one has to resort to approximate formulas and numerical methods (e.g., Barton 1995; Iwasa et al. 2003, 2004b; Serra and Haccou 2007). However, in our special case, a recursive solution can be derived and readily permits the calculation of the introgression probability for any given allele configuration.

We find that both linked and unlinked deleterious alleles can significantly hamper the introgression of an adaptive allele. However, the characteristic of this barrier depends on whether linkage between the beneficial and the deleterious alleles is loose or tight. Loosely linked and unlinked deleterious alleles reduce the introgression probability by a factor that is roughly independent of the strength of the beneficial allele. In this parameter range, our results are analogous to those of Bengtsson (1985) and Barton and Bengtsson (1986), who derive a so-called gene-flow factor or barrier strength to describe the effect of a genetic barrier on the flow of a neutral marker allele (compare Appendix B). The relative reduction due to a single loosely linked allele is approximately $s_{\text {del}}/r$, where $s_{\text {del}}$ is the deleterious effect and $r$ the recombination probability (see Eq. (50), Sect. 6.2). Strongly deleterious alleles ($s_{\text {del}} > 0.05$) can have a substantial effect even if unlinked ($r=0.5$). In agreement with Bengtsson (1985), we find that the influence of several unlinked deleterious alleles is well approximated by a single deleterious allele of the compound effect [Eq. (45)]. The simple picture of a gene-flow factor holds for $r \gg s_{\text {ben}}$ (where $s_{\text {ben}}$ is the effect of the beneficial allele), but breaks down for tighter linkage. For small $r$, the relative reduction scales as $1 - r/(|s_{\text {ben}}-s_{\text {del}}|)$ and thus depends explicitly on the strength of the beneficial allele [cf. Eq. (49)].

Our results on adaptive gene introgression can also be compared with results by Barton (1995) on the reduction of the fixation probability of a new beneficial mutation due to interference with standing deleterious variation. Barton (1995) assumes that the deleterious alleles segregate under mutation-selection balance in the population when the advantageous allele appears. Consider first the limiting case where a deleterious allele segregates at a single locus with frequency $u$. The beneficial mutation can arise on a genome that does or does not carry the deleterious allele. Technically, the introgression probability in our model corresponds most closely to the fixation probability of the beneficial mutation given that it arises on a genome with the deleterious allele [denoted by $P_u$ in Barton (1995)]. In that case, its fixation probability can be significantly reduced. However, the reduction due to segregating deleterious variation is generally much weaker than in our case, where both alleles enter the population via a single introgression event. This is because relative fitness of the double mutant is higher if the deleterious allele segregates in the population. A numerical comparison confirms that the result $P_u/(2\sigma _{(0,0)})$ as given by Eq. (16) and (17a) in Barton (1995) converges to $(1-Q_{(0,1)})/\sigma _{(0,0)}$ [cf. Eq. (47c)] as the mutation rate and hence the frequency of the deleterious allele tend to zero. (Note that the results in Barton (1995) are based on a Poisson distribution of the offspring number such that the establishment probability of an isolated beneficial allele is $2\sigma _{(0,0)}$ while it is $\sigma _{(0,0)}$ in our model). Our results for the introgression probability hence represent limiting cases of the results in Barton (1995) ($P_u$ in the limit $u\rightarrow 0$). However, for more than one deleterious allele, the equations in Barton (1995) do not allow for an analytical solution, while this is possible for the case of introgression, as shown by our results. The total fixation probability in the segregating-alleles case is a weighted average of the cases that the beneficial allele appears on a genome with and without the deleterious allele; the weighting factor depends on the mutation rate. This can lead to widely diverging conclusions as compared to the introgression scenario. For example, for $\sigma _{(0,1)}<0$ and complete linkage, the weighted fixation probability of the beneficial mutation is reduced by $u$ relative to its value in absence of the deleterious allele [Barton 1995, Eq. (17b)]. In contrast, the introgression probability (as well as $P_u$) are zero in that case. For $\sigma _{(0,1)}>0$, the relative reduction is at most $u(s_{\text {del}}/{\sigma _{(0,0)}})^2$, i.e., much smaller than for introgression ($s_{\text {del}}/{\sigma _{(0,0)}}$). Note that the reduction in the weighted fixation probability is caused by the recurrent generation of deleterious alleles that appear on genomes carrying the adaptation. The presence of deleterious alleles itself even slightly increases the weighted fixation probability [this term is very small and neglected in Barton (1995)]. Finally, Barton (1995) finds that for two loci flanking the beneficial mutation, the effects of the two deleterious alleles approximately multiply. This does not hold true for the different biological scenario of adaptive gene introgression.

Linked deleterious alleles can render successful introgression after a single hybridization event extremely unlikely. In order to assess whether even introgression probabilities of the order of $10^{-8}-10^{-6}$ are still evolutionary relevant, it is helpful to compare these values to the probability of adaptation by de-novo mutations. With a point mutation probability of $\sim 10^{-8}$ and a selective advantage of $1\,\%$, the probability that a specific mutation occurs in a specific individual and thereafter rises to fixation, is $\sim 10^{-10}$. For complex adaptations, the probability is even lower. Depending on the probability of hybridization, adaptive gene introgression can hence be a relevant evolutionary process. Hybridization rates are potentially high, and even if the success probability of each single hybridization event is low, the probability that any hybridization event is followed by adaptive gene introgression is appreciable. This consideration is particularly important in an agricultural context where (genetically modified) crops grow next to wild plants in large areas all over the world for many years. Gosh and Haccou (2010) and Gosh et al. (2012a, b) therefore suggest the so-called hazard rate as a measure for risk assessment, as the hazard rate takes both the hybridization rate and the introgression probability into account.

*The hitchhiking probability* Weakly deleterious alleles that are closely linked to the adaptive allele can hitchhike to fixation. We developed a framework to estimate which haplotype finally fixes in the population, depending on the alien haplotype that was originally introduced. The approach is based on a split of the process into two phases: the establishment phase of the adaptive allele and the sweep during which further deleterious alleles can be lost. What is the respective relevance of the stochastic and the deterministic phase in this scenario? In the simplest case, there are only two loci under selection: one locus with the advantageous and one locus with a deleterious allele that can hitchhike to fixation. We can then distinguish two parameter regimes: if the product of the selection coefficient and the population size, $N s_{\text {del}}$, is of order $1$ or smaller, the impact of the stochastic phase is significant. The probability for the hitchhiker to survive this phase depends strongly on the selection coefficients of both the beneficial and the deleterious allele, and on the recombination rate, but is independent of the population size ($1-r s_{\text {ben}}/(s_{\text {ben}}-s_{\text {del}})^2$, cf. Eq. (54)). However, if the product of selection and population size is large, the stochastic phase can be ignored and the behavior is dominated by the deterministic phase. Hitchhikers will survive this phase if no haplotype without the deleterious allele can establish. Since the duration of the deterministic phase is $\sim 1/(s_{\text {ben}}-s_{\text {del}})$ and the number of successful new recombinants per generation roughly $\sim s_{\text {ben}}Nr$, we see that the hitchhiking probability will strongly depend on $Nr$, while the effect of selection partly cancels. This is confirmed by our more precise calculations (Eq. (40)). The situation is different if additional deleterious alleles render the initial haplotype itself deleterious. In that case, establishment of the adaptation is contingent on the early loss of deleterious alleles, and depending on the allelic configuration, the stochastic establishment phase will have a strong impact on hitchhiking irrespective of the population size. To good approximation, all alleles that cause serious maladaptation and are located on the same side of the beneficial allele can be summarized to a single allele of the compound effect, reducing the dimensionality of the problem. The impact of these additional alleles fades quickly with increasing recombination distance. In contrast to introgression, unlinked alleles have no visible effect on the hitchhiking probability conditioned on successful introgression. These insights essentially generalize to more than one possible deleterious hitchhiker.

Hartfield and Otto (2011) analyze the hitchhiking probability of a single deleterious allele in the absence of other deleterious alleles. They present two approaches to the problem: a semi-deterministic approach based on branching process theory (which also serves as the basis for our analysis of the deterministic phase), and a diffusion approach. In both cases, however, they condition on establishment of type $(0,1)$. Their analysis thus ignores aspects of the stochastic establishment phase and consequently applies only to the regime of large $Ns_{\text {del}}$.

*Selection and recombination in the introgression process* Summarizing, we can identify three fundamentally different genomic scales in units of the recombination rate that matter for adaptive gene introgression. First, deleterious alleles on the introgression haplotype affect the introgression probability of the beneficial allele across distances on the order of the deleterious selection coefficient ($r \sim s_{\text {del}}$; cf. Eq. (50)). Strongly deleterious alleles thus still matter even if they are unlinked. Importantly, the absolute strength of selection is crucial for the failure or success of introgression. For the hitchhiking probability of a single deleterious allele, we find two relevant scales that stem from the stochastic and the deterministic phases of the hitchhiking process, respectively. In the stochastic phase, this scale is set by the selection coefficient of the haplotype containing both the beneficial and the deleterious allele ($r \sim s_{\text {ben}}-s_{\text {del}}$; cf. Eq. (54)). Similar to the introgression probability it is the strength of selection that matters. In contrast, for the deterministic phase, the scale is primarily set by the inverse population size ($r \sim 1/N$). The rate of loss of the deleterious allele during the deterministic phase is also affected by selection. However, in contrast to the stochastic phase, only the ratio of selection coefficients $(s_{\text {del}}/s_{\text {ben}}$) enters and not the absolute strength [Eq. (40)]. Usually (but not always), effects from the deterministic phase are relevant already over much shorter distances than effects from the stochastic phase and will therefore dominate. Finally, a third scale for the distance $r$ between the beneficial allele and a deleterious hitchhiker becomes relevant if hitchhiking is compromized by further deleterious alleles in the genomic background. For cases where an additional deleterious allele at distance $R$ to the hitchhiking allele suppresses the Malthusian fitness of that original introgression haplotype below zero, we find that the relevant scale for hitchhiking success is primarily set by the relative size of the recombination rates, i.e., $r \sim R$ [cf. Eqs. (56), (60)].

**Fig. 12:**
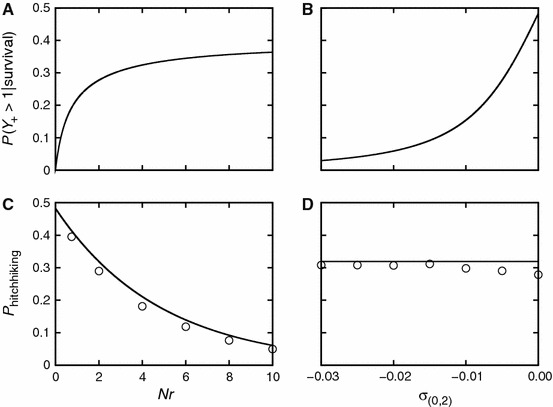
Panels **a** and **b**: The probability that more than one successful recombination event takes place, $P(Y_+>~1|\text {survival})$ [see Eq. (151)] as a function of $r$ and $\sigma _{(0,2)}$, respectively. Panels **c** and **d**: The hitchhiking probability of the deleterious allele as a function of $r$ and $\sigma _{(0,2)}$. Parameter values are: $I=0$, $J=2$, $\sigma _{(0,0)}=0.075$, $\sigma _{(0,1)}=0.07$, $N=$10,000, $r_{(0,0)}^{(0,2)}=r_{(0,1)}^{(0,2)}=r$, $\sigma _{(0,2)}=-0.005$ (Panels **a** and **c**), $Nr=2$ (Panels **b** and **d**). The *circles* denote simulation results. Each simulation point is the average of $2\cdot 10^4$ successful introgression events

**Fig. 13:**
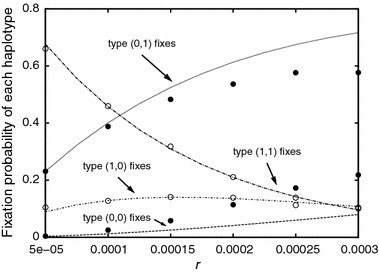
The hitchhiking probability of linked alleles as a function of recombination. Parameter values are: $I=J=1$, $\sigma _{(0,0)}=0.075$, $\sigma _{(0,1)}=0.07$, $\sigma _{(1,0)}=0.06$, $N=10,000$, $r^{(1,1)}_{(0,1)}= r^{(1,1)}_{(1,0)}$. The *circles* denote simulation results. Each simulation point is the average of 2,000 successful introgression events

*Limitations and extensions* The mathematical analysis of the model has two major restrictions. The first one is the common constraint of branching process approximations for establishment probabilities of beneficial alleles: for small populations, the branching process approach underestimates the true probability. In particular, our multitype branching process can contain supercritical, critical, and subcritical types. If the population size is small, fixation of deleterious haplotypes by genetic drift can be more likely than survival of the branching process. The second restriction concerns the analysis of the hitchhiking probability. The derived approximations rely on the assumption that initially, a single haplotype establishes and starts sweeping before haplotypes with fewer deleterious alleles possibly establish. In particular when linkage is not tight, this assumption is not necessarily satisfied. However, this introduces only a small error, and the approximation usually still yields good results (cf. Fig. 12). Relaxing the assumption would require the assessment of the stochastic dynamics of two or more types that simultaneously establish in the population at different random speeds, which would severely complicate the calculations. The analysis of the deterministic phase increases in complexity with the number of positively selected haplotypes. In order to keep it tractable, we assume that at most two successful recombinant haplotypes are generated during the fixation process of the adaptive allele. While this assumption is again well justified for tight linkage, it leads to strong deviations as recombination gets stronger (cf. Fig. 13).

The model assumes a population of haploid individuals or diploids without dominance. The results for the introgression probability can be directly generalized to apply to diploids with dominance unless the beneficial allele is completely recessive: as long as introgressed alleles are rare in the population, they only appear in heterozygotes. Equation (2) therefore still applies if fitness refers to heterozygote fitness. As soon as the introgressed alleles become more frequent, both copies of an individual’s chromosome might carry introgressed material. The sweep of a selectively favored haplotype is therefore strongly altered if the alleles are not co-dominant. The length and shape of this frequency path is, however, crucial for the hitchhiking probability. Our approach can be extended by this element (see Hartfield and Glémin (2014) for a single deleterious hitchhiker if there is dominance). The model furthermore assumes that selection against the deleterious alleles is independent of the genetic background. Recombination therefore increases the fitness of later generations of hybrids. However, by recombination, incompatibilities can be generated. In that case, later hybrids display a lower fitness than early generation hybrids until further recombination removes the incompatible alleles.

Beyond these genetic confinements, the second class of model extensions concerns the ecological setting. The model assumes that the alien genome arrives by long range dispersal in a panmictic population. An important extension, which would considerably affect the results, is the incorporation of spatial structure. A very different situation from the one analyzed in this paper arises if dispersal is local and strong and recurrent gene flow builds up a hybrid zone. Under these circumstances, as discussed in Barton (1979), a single-locus cline does not significantly hamper the spread of a beneficial allele from one population to the other.

To summarize, we set up a minimal model in order to reveal fundamental principles that are effective in the introgression process of a favorable allele. In particular, the analysis helps to build an intuitive understanding of how deleterious alleles impact adaptive gene introgression.

## Figures and Tables

**Fig. 10:**
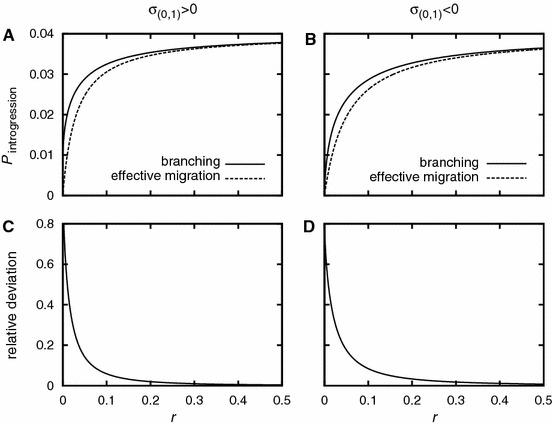
Panels **a** and **b**: The introgression probability as a function of recombination. The *solid line* is based on the branching process approach, Eq. (2). The *dashed line* is obtained by Eq. (75). Panels **c** and **d**: The relative error between the approximations $(1-Q_{(0,1)}- P_{\text {introgression}}^{m_\text {eff}})/(1-Q_{(0,1)})$. The parameter values are: $I=0$, $J=1$, $\sigma _{(0,0)}=0.04$, $\sigma _{(0,1)}=0.01$ (Panels **a** and **c**) or $\sigma _{(0,1)}=-0.01$ (Panels **b** and **d**)

**Fig. 11:**
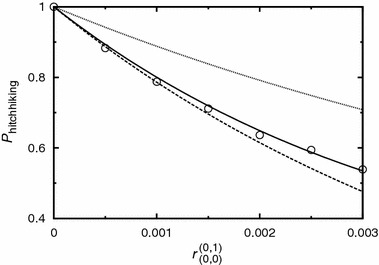
The hitchhiking probability of a linked deleterious allele as a function of the recombination probability. The *solid line* is based on Eq. (53) as in Fig. 4. The *dashed line* shows $P_{(0,1)}^{(0,1)}P^*(1/N)$ where $P_{(0,1)}^{(0,1)}$ and $P^*$ are given by Eq. (52) and the solution of Eq. (148), respectively. The *dotted line* represents $P^*(1/N)$. The parameter values are the same as for Fig. 4D. For $N=$10,000 as in Fig. 4C, both results are basically indistinguishable

## Appendix A: Proof of Theorem 1

We can reinterprete the branching process as follows: An individual of type $(i,j;f)$ “dies” at rate $2-\sigma _{(i,j;f)}$ and at death, it produces either zero or two offspring, one of which is of its own type. We now consider the embedded discrete-time process:

- With probability $\frac{1-\sigma _{(i,j;f)}}{2-\sigma _{(i,j;f)}}$, it has $0$ offspring.
- With probability $$\left( {\begin{array}{c}f\\ g\end{array}}\right) \left( \frac{1}{2}\right) ^f \frac{1-r_{(i,j)}}{2-\sigma _{(i,j;f)}}$$, it produces an offspring of its own type and an offspring of type $(i,j;g)$ with $g\le f$.
- With probability $$\left( {\begin{array}{c}f\\ g\end{array}}\right) \left( \frac{1}{2}\right) ^f \frac{r^{(i,j)}_{(i,k)}}{2-\sigma _{(i,j;f)}}$$, it produces an offspring of its own type and an offspring of type $(i,k;g)$ with $g\le f$ and $k<j$.
- With probability $$\left( {\begin{array}{c}f\\ g\end{array}}\right) \left( \frac{1}{2}\right) ^f \frac{r^{(i,j)}_{(k,j)}}{2-\sigma _{(i,j;f)}}$$, it produces an offspring of its own type and an offspring of type $(k,j;g)$ with $g\le f$ and $k<i$.

Within this scheme, the offspring generating function of an individual of type $(i,j;f)$ is given by

63 $$\begin{aligned} G^{(i,j;f)}(\mathbf {s})&=\left( \frac{1}{2}\right) ^f\! \frac{1-r_{(i,j)}}{2-\sigma _{(i,j;f)}} s_{(i,j;f)}^2 \nonumber \\&\quad +\sum \limits _{g=0}^{f} \left( {\begin{array}{c}f\\ g\end{array}}\right) \left( \frac{1}{2}\right) ^f\! \left\{ \sum \limits _{k=0}^{i-1} \frac{r^{(i,j)}_{(k,j)}}{2-\sigma _{(i,j;f)}} s_{(k,j;g)} \!+\! \sum \limits _{k=0}^{j-1} \frac{r^{(i,j)}_{(i,k)}}{2-\sigma _{(i,j;f)}} s_{(i,k;g)}\right\} s_{(i,j;f)} \nonumber \\&\quad + \sum \limits _{g=0}^{f-1} \left( {\begin{array}{c}f\\ g\end{array}}\right) \left( \frac{1}{2}\right) ^f \frac{1-r_{(i,j)}}{2-\sigma _{(i,j;f)}} s_{(i,j;g)}s_{(i,j;f)}+\frac{1-\sigma _{(i,j;f)}}{2-\sigma _{(i,j;f)}}, \end{aligned}$$

where $\mathbf {s}$ is a vector with elements $\left\{ s_{(k_1,k_2;g)}; 0\le k_1 \le I, 0\le k_2 \le J, 0\le g\le F\right\}$. Let $\mathbf {G}(\mathbf {s})$ be the vector whose components are the offspring generating functions of all possible types. According to the general theory of multitype branching processes, the extinction probability is given by the root of

64 $$\begin{aligned} \mathbf {G}(\mathbf {s})=\mathbf {s} \end{aligned}$$

in the unit cube, which is closest to the origin (Sewastjanow 1974 p. 115).

First note that Eq. (64) is equivalent to Eq. (3) (identifying the solution of Eq. (64) with the vector with elements $\big \{Q_{(k_1,k_2;g)}; 0\le k_1 \le I, 0\le k_2 \le J, 0\le g \le F\big \}$).

Equation (64) can be solved recursively starting with type $(0,0;0)$, for which we obtain

65 $$\begin{aligned} Q_{(0,0;0)}=1-\sigma _{(0,0;0)}<1. \end{aligned}$$

Following Sewastjanow (1974), in each subsequent step of the recursion, there exists a unique solution $<1$. It remains to show that we obtain this solution if we solve the quadratic equation for the smaller root. We use the following abbreviations:

66 $$\begin{aligned} \Sigma _g&:= \sum \limits _{k=0}^{j-1} r^{(i,j)}_{(i,k)}Q_{(i,k;g)} + \sum \limits _{k=0}^{i-1} r^{(i,j)}_{(k,j)}Q_{(k,j;g)}, \end{aligned}$$

67 $$\begin{aligned} \Sigma&:= \left( \frac{1}{2}\right) ^f \left\{ \sum \limits _{g=0}^{f-1}\left( {\begin{array}{c}f\\ g\end{array}}\right) (1-r_{(i,j)})Q_{(i,j;g)}+\sum \limits _{g=0}^{f} \left( {\begin{array}{c}f\\ g\end{array}}\right) \Sigma _g\right\} , \end{aligned}$$

68 $$\begin{aligned} a&:= \left( \frac{1}{2}\right) ^f(1-r_{(i,j)})>0, \end{aligned}$$

69 $$\begin{aligned} b&:= -(1-\sigma _{(i,j;f)}+1-\Sigma ), \end{aligned}$$

70 $$\begin{aligned} c&:= 1-\sigma _{(i,j;f)}\ge 0. \end{aligned}$$

Note that $$r\ge \Sigma _g, \forall g$$.

With these abbreviations, the two roots are given by

71 $$\begin{aligned} q_1&= \frac{-b+\sqrt{(b^2-4ac)}}{2a}, \end{aligned}$$

72 $$\begin{aligned} q_2&= \frac{-b-\sqrt{(b^2-4ac)}}{2a}. \end{aligned}$$

We first prove that both roots are real and $\ge 0$. As a preliminary step, we show that $(1-\Sigma ) \ge \left( \frac{1}{2}\right) ^f(1-r_{(i,j)})$:

73 $$\begin{aligned} 1-\Sigma&= 1 - \left( \frac{1}{2}\right) ^f \left\{ \sum \limits _{g=0}^{f-1}\left( {\begin{array}{c}f\\ g\end{array}}\right) (1-r_{(i,j)})Q_{(i,j;g)}+\sum \limits _{g=0}^{f}\left( {\begin{array}{c}f\\ g\end{array}}\right) \Sigma _g\right\} \nonumber \\&\ge 1 - \left( \frac{1}{2}\right) ^f \left\{ \sum \limits _{g=0}^{f-1}\left( {\begin{array}{c}f\\ g\end{array}}\right) (1-r_{(i,j)})+\sum \limits _{g=0}^{f} \left( {\begin{array}{c}f\\ g\end{array}}\right) \Sigma _g\right\} \nonumber \\&= 1-\left( \frac{1}{2}\right) ^f \left\{ \sum \limits _{g=0}^f \left( {\begin{array}{c}f\\ g\end{array}}\right) \left[ (1-r_{(i,j)}) +\Sigma _g \right] -(1-r_{(i,j)}) \right\} \nonumber \\&\ge 1-\left\{ \sum \limits _{g=0}^f \left( {\begin{array}{c}f\\ g\end{array}}\right) \left( \frac{1}{2}\right) ^f -(1-r_{(i,j)})\left( \frac{1}{2}\right) ^f \right\} \nonumber \\&= (1-r_{(i,j)})\left( \frac{1}{2}\right) ^f. \end{aligned}$$

Notice: $b<0$ since $\sigma _{(i,j;f)}\le 1$ and $1-\Sigma \ge (1-r_{(i,j)})\left( \frac{1}{2}\right) ^f>0$.

We now show that $b^2 - 4ac \ge 0$.

74 $$\begin{aligned} b^2-4ac&= (1-\sigma _{(i,j)}+1-\Sigma )^2 - 4(1-\sigma _{(i,j)})(1-r_{(i,j)})\left( \frac{1}{2}\right) ^f \nonumber \\&\ge (1-\sigma _{(i,j)}+1-\Sigma )^2 - 4(1-\sigma _{(i,j)})(1-\Sigma )^2 \nonumber \\&= (1-\sigma _{(i,j)}-(1-\Sigma ))^2 \ge 0, \end{aligned}$$

where we have used that $(1-r_{(i,j)})\left( \frac{1}{2}\right) ^f\le 1-\Sigma$. Since $a>0$, we conclude that both roots are positive.

It remains to prove that $q_2 \le 1$. Note that $\mathbf {1}$ is a root of the equation since $\mathbf {G}(\mathbf {1})=\mathbf {1}$. We furthermore see that $q_2$ is a decreasing function of $\Sigma$ and thus of all $Q_{(k,j)}$ and $Q_{(i,k)}$, if $c>0$. For $c=0$, it holds that $q_2=0$. It is hence clear that $q_2 \le 1$. From Sewastjanow (1974), we even know that $q_2 < 1$ (because $Q_{(0,0;0)}<1$). $\square$

Note that instead of considering the embedded discrete time process we could have directly resorted to the corresponding result for the extinction probability of the continuous time processes (Sewastjanow 1974, p. 116), but it seemed more illustrative in this way.

## Appendix B: Approximation of the establishment probability via an effective migration rate

Bengtsson (1985) and Barton and Bengtsson (1986) determine by how much deleterious alleles hamper the flow of a neutral marker locus from one population into another, where Bengtsson (1985) mainly focuses on unlinked and Barton and Bengtsson (1986) on linked deleterious alleles. The impact is expressed via a so-called gene flow factor, which is given by the ratio of an effective migration rate $m_e$ and the actual migration rate $m$ (Eqs. (1), (3), and (5) in Bengtsson (1985); Eqs. (A3) and (A7) in Barton and Bengtsson (1986); the inverse of the gene flow factor gives the barrier strength to gene flow). Building on these results for the introgression of neutral marker loci, the introgression probability of a single adaptive allele with selective advantage $s$ can be approximated by

75 $$\begin{aligned} P_{\text {introgression}}^{m_\text {eff}}=\frac{m_e}{m} s. \end{aligned}$$

Figure 10 shows how this approximation compares to the establishment probability as obtained by the branching process approach if there is just one deleterious allele. The deviation is large for tight linkage and gets small as the recombination distance between the loci increases; for lose linkage, it is negligible. This observation generalizes to scenarios with more deleterious alleles. Deviations for tight linkage are to be expected: As an example, consider the basic scenario $I=0$, $J=1$, and $s_{\text {del}}<\sigma _{(0,0)}$. For $r=0$, the introgression probability of the adaptive allele equals $\sigma _{(0,0)}-s_{\text {del}}$. A neutral marker allele that is equally tightly linked, however, establishes with probability $\approx 0$ (within the approximations by Bengtsson (1985) and Barton and Bengtsson (1986), its establishment probability equals zero; in a finite population, it might fix by drift). We now turn to the other extreme, i.e., when the deleterious alleles are all unlinked. We can perform a first-order Taylor expansion in $s_{\text {del}}$ of the result for $m_e/m$ with $I=0$, $F=1$ as found by Bengtsson (1985)

76 $$\begin{aligned} \frac{m_\text {e}}{m} = 1-2s_{\text {del}} + \mathcal {O}(s_{\text {del}}^2). \end{aligned}$$

This is in agreement with Eq. (46). Bengtsson (1985) furthermore finds that to good approximation, the compound effect of all deleterious alleles, not their individual effects, matters. Again, this accords with our findings [cf. Sect. 6.1].

## Appendix C: Proof of Eq. (32)

We prove that within approximation (30), it holds

77 $$\begin{aligned} \sum \limits _{i,j} P_{(i,j)}^{(I,J)} =1, \end{aligned}$$

where $P_{(\cdot , \cdot )}^{(\cdot ,\cdot )}$ is defined by the approximative formula (30). We carry out a proof by induction. Eq. (77) builds our induction hypothesis.

- Base case $(I,J)=(0,0)$: Since $P_{(i,j)}^{(0,0)}=\delta _{i,0}\delta _{j,0}$, it holds $\sum \nolimits _{i,j} P_{(i,j)}^{(0,0)} =1$.
- Inductive step: Let the hypothesis be true for all pairs $(k,m)$ with $k<n$ and $(n,k)$ with $k<m$. We show that it is true for $(n,m)$:
  78 $$\begin{aligned}&\sum _{i,j}P_{(i,j)}^{(n,m)} \nonumber \\&\quad = \frac{\sum \nolimits _{k=0}^{n-1}r^{(n,m)}_{(k,m)} (1-Q_{(k,m)})\sum _{i,j}P_{(i,j)}^{(k,m)}+ \sum \nolimits _{k=0}^{m-1} r^{(n,m)}_{(n,k)} (1-Q_{(n,k)}) \sum _{i,j} P_{(i,j)}^{(n,k)} }{\sum \nolimits _{k=0}^{n-1}r^{(n,m)}_{(k,m)} (1-Q_{(k,m)})+ \sum \nolimits _{k=0}^{m-1} r^{(n,m)}_{(n,k)} (1-Q_{(n,k)})}\nonumber \\&\quad = \frac{\sum \nolimits _{k=0}^{n-1}r^{(n,m)}_{(k,m)} (1-Q_{(k,m)})\cdot 1+ \sum \nolimits _{k=0}^{m-1} r^{(n,m)}_{(n,k)} (1-Q_{(n,k)}) \cdot 1 }{\sum \nolimits _{k=0}^{n-1}r^{(n,m)}_{(k,m)} (1-Q_{(k,m)})+ \sum \nolimits _{k=0}^{m-1} r^{(n,m)}_{(n,k)} (1-Q_{(n,k)}) } =1. \end{aligned}$$
  $\square$ The proof works analogously if the number of unlinked alleles is larger than $0$.

## Appendix D: Unlinked alleles of arbitrary effect

For the main text, we assumed that each unlinked allele has the same selection coefficient. Here, we give the generalization to arbitrary effects.

Let $\mathcal {F}=\left\{ \mathbf{e} = (e_1,e_2,\dots ,e_F); e_i \in \left\{ 0,1\right\} \right\}$. We define $|\mathbf{e}|=\sum \nolimits _{i=1}^{F}e_i$. The set of unlinked deleterious alleles carried by an individual can be characterized by a vector in $\mathcal {F}$ where $1$ and $0$ at a given position denote the presence or absence of a specific deleterious allele. In this notation, the transitions of the branching process read with $|\varvec{e}_f|=f$:

79 $$\begin{aligned} P((i,j;\varvec{e}_f) \rightarrow \emptyset )&= 1-\sigma _{(i,j;\varvec{e}_f)},\\ P((i,j;\varvec{e}_f) \rightarrow \{(i,j;\varvec{e}_f),(i,j;\varvec{e}_g)\})&= \left( \frac{1}{2}\right) ^f (1-r_{(i,j)}) \quad for \quad \varvec{e}_f-\varvec{e}_g \in \mathcal {F},\\ P((i,j;\varvec{e}_f) \rightarrow \{(i,j;\varvec{e}_f),(i,k;\varvec{e}_g)\})&= \left( \frac{1}{2}\right) ^f r^{(i,j)}_{(i,k)} \quad for \quad k<j, \varvec{e}_f-\varvec{e}_g \in \mathcal {F},\\ P((i,j;\varvec{e}_f) \rightarrow \left\{ (i,j;\varvec{e}_f),(k,j;\varvec{e}_g) \right\} )&= \left( \frac{1}{2}\right) ^f r^{(i,j)}_{(k,j)} \quad for \quad k<i, \varvec{e}_f-\varvec{e}_g \in \mathcal {F}.\nonumber \\ \end{aligned}$$

The recursive equation for the extinction probability becomes:

80 $$\begin{aligned}&\left( \frac{1}{2}\right) ^f (1-r_{(i,j)})Q^2_{(i,j;\varvec{e}_f)} \nonumber \\&\quad \quad + \sum \limits _{\varvec{e}_g; \varvec{e}_f-\varvec{e}_g \in \mathcal {F}} \left( \frac{1}{2}\right) ^f \left\{ \sum \limits _{k=0}^{i-1} r_{(k,j)}^{(i,j)} Q_{(k,j;\varvec{e}_g)} + \sum \limits _{k=0}^{j-1} r_{(i,k)}^{(i,j)} Q_{(i,k;\varvec{e}_g)} \right\} Q_{(i,j;\varvec{e}_f)}\nonumber \\&\quad \quad + \sum _{\begin{array}{c} \varvec{e}_g;\varvec{e}_f-\varvec{e}_g \in \mathcal {F},\nonumber \\ \varvec{e}_f-\varvec{e}_g \ne \varvec{0} \end{array}} \left( \frac{1}{2}\right) ^f (1-r_{(i,j)}) Q_{(i,j;\varvec{e}_g)} Q_{(i,j;\varvec{e}_f)} - (2-\sigma _{(i,j;\varvec{e}_f)})Q_{(i,j,\varvec{e}_f)} \nonumber \\&\quad \quad + 1 - \sigma _{(i,j;\varvec{e}_f)}=0. \end{aligned}$$

The proof is analogous to before.

Similarly, we can generalize approximation Eq. (34):

81 $$\begin{aligned} P_{(i,j;\varvec{0})}^{(I,J;\varvec{e}_F)}&= \frac{\sum \limits _{\varvec{e}_l} \left( \frac{1}{2}\right) ^F\! A_{\varvec{e}_l} +\sum \limits _{\varvec{e}_l \ne \varvec{e}_F}\left( \frac{1}{2}\right) ^F(1-r_{(I,J)})(1-Q_{(I,J;\varvec{e}_l)})P_{(i,j;\varvec{0})}^{(I,J;\varvec{e}_l)}}{\sum \limits _{\varvec{e}_l}\left( \frac{1}{2}\right) ^F B_{\varvec{e}_l}+\sum \limits _{\varvec{e}_l \ne \varvec{e}_F}\left( \frac{1}{2}\right) ^F(1-r_{(I,J)})(1-Q_{(I,J;\varvec{e}_l)})}. \nonumber \\ A_{\varvec{e}_l}&= \sum \limits _{i=0}^{I-1}r^{(I,J)}_{(i,J)} (1-Q_{(i,J;\varvec{e}_l)})P_{(i,j;\varvec{0})}^{(i,J;\varvec{e}_l)}+ \sum \limits _{j=0}^{J-1} r^{(I,J)}_{(I,j)} (1-Q_{(I,j;\varvec{e}_l)})P_{(i,j;\varvec{0})}^{(I,j;\varvec{e}_l)} \nonumber \\ B_{\varvec{e}_l}&= \sum \limits _{i=0}^{I-1}r^{(I,J)}_{(i,J)} (1-Q_{(i,J;\varvec{e}_l)})+ \sum \limits _{j=0}^{J-1} r^{(I,J)}_{(I,j)} (1-Q_{(I,j;\varvec{e}_l)}) \end{aligned}$$

For $\left( \frac{1}{2}\right) ^F(1-r_{(I,J)})-(1-\sigma _{(I,J;F)})>0$, we approximate:

82 $$\begin{aligned} P_{(i,j;\varvec{0})}^{(I,J;\varvec{e}_F)}=\delta _{I,i}\delta _{J,j}. \end{aligned}$$

## Appendix E: General lemmata

Here we summarize some general lemmata which we use in the derivation of the hitchhiking probability. Although the lemmata either are not new or follow immediately from known results, we briefly sketch the proofs.

We consider a multitype branching process with $N+1$ types on $(\Omega , \mathcal {F}, P)$. The number of individuals of type $i$ ($i=0,1,\dots ,N$) in generation $n$ is $Z_n^{(i)}$. Let $Z_0^{(N)}=1$ and $Z_0^{(i)}=0$ if $i\ne N$, i.e., the process is started by an individual of type $N$; the term “generation” refers to the distance to this founding individual. Individuals of type $i$ reproduce at rate $1$ and die at rate $1-\sigma _i$. Let the offspring of a type $N$ individual be of type $i$ with probability $r_i$. If a type $N$ individual gives birth to an individual of type $i<N$, we call this a recombination event from type $N$ to type $i$. We define $r:= \sum \nolimits _{k=0}^{N-1} r_k = 1-r_N$.

### **Lemma 2**

The probability generating function (p.g.f.) $f(s)$ of the offspring distribution of a type $N$ individual is given by

83 $$\begin{aligned} f(s)=\frac{1-p}{1-ps} \quad \text {with} \quad p=\frac{1}{2-\sigma _N}. \end{aligned}$$

### *Proof*

Individuals have a geometric offspring distribution; for the random offspring number $X$ of a type $N$ individual, it holds:

84 $$\begin{aligned} P(X=k) = \left( \frac{1}{2-\sigma _N}\right) ^k \frac{1-\sigma _N}{2-\sigma _N}, \end{aligned}$$

and therefore

85 $$\begin{aligned} f(s)&= \sum _{k=0}^{\infty } P(X=k) s^k = \sum _{k=0}^{\infty } \left( \frac{1}{2-\sigma _N}\right) ^k \frac{1-\sigma _N}{2-\sigma _N} s^k \nonumber \\&= \frac{1-\sigma _N}{2-\sigma _N} \frac{1}{1-\frac{s}{2-\sigma _N}} = \frac{1-p}{1-ps}. \end{aligned}$$

$$\square $$

### **Lemma 3**

The joint p.g.f. of $(Z_1^{(0)},Z_1^{(1)},\dots ,Z_1^{(N)})$ is given by

86 $$\begin{aligned} F(s_0,s_1,\dots ,s_N) = f(r_0s_0+r_1s_1+\dots r_Ns_N). \end{aligned}$$

### *Proof*

We restrict to $N=1$; the generalization to larger $N$ is straightforward. Let $p(k)$ be the probability that an individual of type $1$ has $k$ offspring and $p(k_0,k_1)$ the probability that it has $k_0$ offspring of type $0$ and $k_1$ offspring of type $1$. It holds (cf. Serra 2006):

87 $$\begin{aligned} F(s_0,s_1)&= \sum \limits _{k_0,k_1} p(k_0,k_1) s_0^{k_0} s_1^{k_1} = \sum \limits _{k=0}^{\infty }\sum \limits _{k_0=0}^{k} \underbrace{ p(k_0,k-k_0) }_{=p(k)\left\{ r_0^{k_0}(1-r_0)^{k-k_0} \left( {\begin{array}{c}k\\ k_0\end{array}}\right) \right\} }\!\!\!\!\!s_0^{k_0}s_1^{k-k_0} \nonumber \\&= \sum \limits _{k=0}^{\infty } p(k) \underbrace{\sum \limits _{k_0=0}^{k} \left( {\begin{array}{c}k\\ k_0\end{array}}\right) (r_0s_0)^{k_0} (s_1(1-r_0))^{k-k_0}}_{=(r_0s_0+(1-r_0)s_1)^k}\nonumber \\&= \sum \limits _{k=0}^{\infty } p(k) (r_0s_0+(1-r_0)s_1)^{k} =f(r_0s_0+(1-r_0)s_1). \end{aligned}$$

$$\square $$

### **Lemma 4**

The extinction probability $q_N$ of type $N$ is given by

88 $$\begin{aligned} q_N = \left\{ \begin{array}{lll}\frac{1-\sigma _N}{1-r} &{} \text {if} &{} \sigma _N > r, \\ 1 &{} \text {if} &{} \sigma _N \le r.\end{array} \right. \end{aligned}$$

### *Proof*

The p.g.f. for the number of offspring of its own type is given by $f(r+(1-r)s)$. From the general theory of single type branching processes, it follows that $q_N$ is the smallest root of

89 $$\begin{aligned} q_N=f(r+(1-r)q_N). \end{aligned}$$

in $$[0,1]$$.

### **Lemma 5**

Let the offspring of type $i$ be of type $j\le i$ only. Consider the process on the set $B=\left\{ \omega : Z_n^{(N)}(\omega ) \rightarrow 0 \quad \text {as} \quad n \rightarrow \infty \right\}$, i.e., the set of paths were type $N$ goes extinct. The probability of the event $B$ is denoted by $q_N$. The joint p.g.f. of $(Z_1^{(0)},Z_1^{(1)},\dots ,Z_1^{(N)})$ in the conditioned process is given by

90 $$\begin{aligned} \hat{F}(s_0,s_1,\dots ,s_N) = \frac{F(s_0,s_1,\dots ,q_Ns_{N})}{q_N}. \end{aligned}$$

### *Proof*

For simplicity, we stick to $N=1$. The generalization to $N>1$ is straightforward. It holds (cf. Athreya and Ney 1972, p. 52f) :

91 $$\begin{aligned} \hat{F}(s_0,s_1)&= \sum \limits _{k_0=0}^{\infty } \sum \limits _{k_1=0}^{\infty } P(Z_1^{(0)}=k_0,Z_1^{(1)}=k_1|B) s_0^{k_0} s_1^{k_1} \nonumber \\&= \frac{\sum \limits _{k_0=0}^{\infty } \sum \limits _{k_1=0}^{\infty } P(Z_1^{(0)}=k_0,Z_1^{(1)}=k_1,B) s_0^{k_0} s_1^{k_1} }{q_1} \nonumber \\&= \frac{\sum \limits _{k_0=0}^{\infty } \sum \limits _{k_1=0}^{\infty } P(Z_1^{(0)}=k_0,Z_1^{(1)}=k_1)q_1^{k_1} s_0^{k_0} s_1^{k_1} }{q_1}\nonumber \\&= \frac{F(s_0,q_1s_1)}{q_1}. \end{aligned}$$

$$\square $$

### **Lemma 6**

Let the offspring of type $i$ be of type $j\le i$ only. Condition the process on extinction of type $N$. The total number of recombination events from type $N$ individuals to other types in the whole process is determined by the p.g.f. $\hat{h}(s)$ with

92 $$\begin{aligned} \hat{h}(s) = \frac{1-psr - \sqrt{(1-psr)^2-4(1-p)p(1-r)}}{2pq_N(1-r)}. \end{aligned}$$

### *Proof*

We first proof the relation

93 $$\begin{aligned} \hat{h}(s) = \hat{F}(s,\hat{h}(s)). \end{aligned}$$

(cf. Serra 2006). Let $\tilde{p}(k)$ be the probability that the total number of recombination events is $k$ and $p(k_r,k_N)$ be the probability that an individual of type $N$ has $k_N$ offspring of type $N$ and $k_r$ offspring of other types. First, note that:

94 $$\begin{aligned} \tilde{p}(k) = \sum \limits _{k_{N=0}}^{\infty } \sum _{j=0}^{k} p(k-j,k_N) \tilde{p}(j|k_N). \end{aligned}$$

Furthermore:

95 $$\begin{aligned} \sum \limits _{k=0}^{\infty }\sum \limits _{k_{N=0}}^{\infty } \sum \limits _{j=0}^k p(k-j,k_N)s^k = \sum \limits _{k_r, k_N} p(k_r,k_N) \sum \limits _{j} \tilde{p}(j|k_N)s^{k_r+j}. \end{aligned}$$

Using this:

96 $$\begin{aligned} \hat{h}(s)&= \sum \limits _{k} \tilde{p}(k) s^k = \sum \limits _{k_r, k_N} p(k_r,k_N) \sum \limits _{j=0}^{\infty } \tilde{p}(j|k_N) s^{j+k_r} \nonumber \\&= \sum \limits _{k_r, k_N} p(k_r,K_N)s^k_r \underbrace{\sum \limits _{j=0}^{\infty } \tilde{p}(j|k_N) s^j}_{=\left( \hat{h}(s)\right) ^{k_N}}. \end{aligned}$$

$$\square $$

It thus holds:

97 $$\begin{aligned} \hat{h}(s)&= \hat{F}(s,\hat{h}(s)) = \frac{F(s,q_N\hat{h}(s))}{q_N} = \frac{f(s r + q_N(1-r)\hat{h}(s))}{q_N}\nonumber \\&= \frac{1}{q_N}\frac{1-p}{1-p(sr+q_N(1-r)\hat{h}(s))}. \end{aligned}$$

This leads to a quadratic equation for $\hat{h}(s)$ which has two solutions. As it must hold that $\hat{h}(1)=1$, we can exclude one of them and obtain Eq. (92). $\square$

If $q_N=1$, we denote the probability generating function by $h(s)$ in the main text.

### **Lemma 7**

With the assumptions of the previous lemma, the joint p.g.f. of the number of recombination events from type $N$ to type $0,1,2,\dots ,N-1$ is given by

98 $$\begin{aligned} h_{1}(s_0,s_1,\dots ,s_{N-1}) = \hat{h}(\frac{r_0}{r}s_0+\dots +\frac{r_{N-1}}{r}s_{N-1}). \end{aligned}$$

### *Proof*

analogous to the proof of Lemma 5.

## Appendix F: The deterministic phase

### Appendix F1: The process is initiated by an individual of type $(0,2)$

We now focus on three types $(0,0)$, $(0,1)$, and $(0,2)$, that all have a positive selection coefficient. All other types are assumed to go extinct. A lineage of type $(0,2)$ starts sweeping. Throughout, we assume that recombination is weak enough that it does not alter the deterministic frequency path of a type.

The deterministic frequency path is given by

99 $$\begin{aligned} x_{(0,2)} = \frac{\bar{\nu }_0 \exp {(\sigma _{(0,2)} t)}}{N-\bar{\nu }_0+\bar{\nu }_0 \exp {(\sigma _{(0,2)} t)}} \end{aligned}$$

with $\bar{\nu }_0=\frac{1}{1-q_{(0,2)}}$ where $q_{(0,2)}$ is defined in Eq. (26) as $q_{(0,2)}=\frac{1-\sigma _{(0,2)}}{1-r_{(0,2)}}$. Recombination events to successful lineages occur at rate

100 $$\begin{aligned} \alpha _{(0,2)}(t) = \alpha _{(0,0)}(t)+\alpha _{(0,1)}(t) \end{aligned}$$

with

101a $$\begin{aligned} \alpha _{(0,0)}(t)&= N x_{(0,2)}(t)(1-x_{(0,2)}(t)) r_{(0,0)}^{(0,2)} p_{\text {est}}^{(0,0)}(x_{(0,2)}(t)), \end{aligned}$$

101b $$\begin{aligned} \alpha _{(0,1)}(t)&= Nx_{(0,2)}(t)(1-x_{(0,2)}(t))r^{(0,2)}_{(0,1)} p_{\text {est}}^{(0,1)}(x_{(0,2)}(t)), \end{aligned}$$

where $p_{\text {est}}^{(0,0)}$ and $p_{\text {est}}^{(0,1)}$ are the establishment probabilities of an individual of type $(0,0)$ and $(0,1)$ respectively and are calculated by suitable substitutions in Eq. (38). We assume in the following that we can set $\bar{\nu }_0/N \approx 0$ in the integration boundaries. Analogous to Eq. (41), the probability that no successful recombination event takes place is then given by

102 $$\begin{aligned} P_{\text {det}}^{((0,2)\rightarrow (0,2))}&\approx \exp {\left[ -\int \limits _0^{\infty } \alpha _{(0,2)}(t)\mathrm {d}t\right] }= \left( \frac{\sigma _{(0,0)}}{\sigma _{(0,0)}-\sigma _{(0,2)}}\right) ^{\!\!-\frac{Nr^{(0,2)}_{(0,0)} \sigma _{(0,0)}(\sigma _{(0,0)}-\sigma _{(0,2)})}{\sigma _{(0,2)}^2}}\nonumber \\&\times \,\, \left( \frac{\sigma _{(0,1)}}{\sigma _{(0,1)}-\sigma _{(0,2)}}\right) ^{\!\!-\frac{Nr^{(0,2)}_{(0,1)} \sigma _{(0,1)}(\sigma _{(0,1)}-\sigma _{(0,2)})}{\sigma _{(0,2)}^2}}. \end{aligned}$$

The probability that no successful type $(0,0)$ is generated up to $t_R$ is given by

103 $$\begin{aligned}&\exp {\left[ -\int \limits _0^{t_R} \alpha _{(0,0)}(t) \mathrm {d}t\right] } \nonumber \\&\quad \approx \exp {\left[ -\!\!\!\!\!\int \limits _{0}^{x_{(0,2)}(t_R)} \!\!\!\!\! Nr^{(0,2)}_{(0,0)} \frac{1}{\sigma _{(0,2)}}\frac{\sigma _{(0,0)} (\sigma _{(0,0)}-\sigma _{(0,2)})}{ (\sigma _{(0,0)}-\sigma _{(0,2)})(1-x) + \sigma _{(0,0)}x} \mathrm {d}x \right] }\nonumber \\&\quad = \left( \frac{\sigma _{(0,0)} - (1 - x_{(0,2)}(t_R))\sigma _{(0,2)}}{\sigma _{(0,0)} - \sigma _{(0,2)}}\right) ^{-\frac{N r_{(0,0)}^{(0,2)}\sigma _{(0,0)}(\sigma _{(0,0)} - \sigma _{(0,2)})}{\sigma _{(0,2)}\sigma _{(0,2)}}} \nonumber \\&\quad \equiv Q_{\text {det}}^{(0,0)}(x_{(0,2)}(t_R)). \end{aligned}$$

The probability that no successful type $(0,1)$ is generated up to time $t_R$ is given by

104 $$\begin{aligned}&\exp {\left[ -\int \limits _0^{t_R} \alpha _{(0,1)}(t) \mathrm {d}t\right] } \nonumber \\&\quad \approx \exp {\left[ -\!\!\!\!\!\int \limits _{0}^{x_{(0,2)}(t_R)} \!\!\!\!\! Nr^{(0,2)}_{(0,1)} \frac{1}{\sigma _{(0,2)}}\frac{\sigma _{(0,1)} (\sigma _{(0,1)}-\sigma _{(0,2)})}{ (\sigma _{(0,1)}-\sigma _{(0,2)})(1-x) + \sigma _{(0,1)}x} \mathrm {d}x\right] }\nonumber \\&\quad = \left( \frac{\sigma _{(0,1)} - (1 - x_{(0,2)}(t_R))\sigma _{(0,2)}}{\sigma _{(0,1)} - \sigma _{(0,2)}}\right) ^{-\frac{N r_{(0,0)}^{(0,2)}\sigma _{(0,1)}(\sigma _{(0,1)} - \sigma _{(0,2)})}{\sigma _{(0,2)}\sigma _{(0,2)}}}\nonumber \\&\quad \equiv Q_{\text {det}}^{(0,1)}(x_{(0,2)}(t_R)). \end{aligned}$$

The probability that as a first event, a successful individual of type $(0,0)$ is generated is given by

105 $$\begin{aligned}&\int \limits _0^{\infty } Q_{\text {det}}^{(0,0)}(x_{(0,2)}(t_R)) Q_{\text {det}}^{(0,1)}(x_{(0,2)}(t_R)) \alpha _{(0,0)}(t_R)\mathrm {d}t_R\nonumber \\&\quad =\int \limits _0^{\infty } Q_{\text {det}}^{(0,0)}(x_{(0,2)}(t_R)) Q_{\text {det}}^{(0,1)}(x_{(0,2)}(t_R))\nonumber \\&\qquad \times N x_{(0,2)}(t)(1-x_{(0,2)}(t))r_{(0,0)}^{(0,2)} p_{\text {est}}^{(0,0)}(x_{(0,2)}(t)) \mathrm {d}t_R\nonumber \\&\quad = \frac{1}{\sigma _{(0,2)}}\int \limits _0^1 Q_{\text {det}}^{(0,0)}(x) Q_{\text {det}}^{(0,1)}(x) N r_{(0,0)}^{(0,2)} p_{\text {est}}^{(0,0)}(x)\mathrm {d}x. \end{aligned}$$

Equivalently, the probability that as a first event, a successful individual of type $(0,1)$ is generated is given by

106 $$\begin{aligned} \frac{1}{\sigma _{(0,2)}}\int \limits _0^1 Q_{\text {det}}^{(0,0)}(x) Q_{\text {det}}^{(0,1)}(x) N r_{(0,1)}^{(0,2)} p_{\text {est}}^{(0,1)}(x)\mathrm {d}x. \end{aligned}$$

In that case, type $(0,1)$ might either rise to fixation, or a successful type $(0,0)$ individual might be generated in the following process. We now calculate the probability of these two events given a type $(0,1)$ individual was generated at time $t_R$. In order to calculate the probability for the generation of a successful type $(0,0)$ individual, we need the frequencies of the three present types (wildtype, type $(0,2)$, and type $(0,1)$) at time $t$ after the recombination event. We have to take into account that in its early phase of growth, the type $(0,1)$ increases faster than predictic by the deterministic path. As before, we therefore replace its initial frequency by the mean of the effective initial population size $\nu$. We assume that during establishment, the type $(0,1)$ individuals exclusively replace wildtype individuals. This assumption has no visible influence on the results. We obtain for the frequencies of wildtype individuals, type $(0,1)$ individuals, and type $(0,2)$ individuals, respectively:

107 $$\begin{aligned}&\tilde{q}(t|x_{(0,2)}(t_R)) \nonumber \\&\quad = \frac{1-x_{(0,2)}(t_R)-\frac{\bar{\nu }(t_R)}{N}}{x_{(0,2)}(t_R)\exp {(\sigma _{(0,2)} t)}+1-x_{(0,2)}(t_R)-\frac{\bar{\nu }(t_R)}{N}+\frac{\bar{\nu }(t_R)}{N}\exp {(\sigma _{(0,1)} t)}},\qquad \end{aligned}$$

108 $$\begin{aligned}&\tilde{x}_{01}(t|x_{(0,2)}(t_R))\nonumber \\&\quad = \frac{\frac{\bar{\nu }(t_R)}{N}\exp {(\sigma _{(0,1)} t)}}{x_{(0,2)}(t_R)\exp {(\sigma _{(0,2)} t)}+1-x_{(0,2)}(t_R)-\frac{\bar{\nu }(t_R)}{N}+\frac{\bar{\nu }(t_R)}{N}\exp {(\sigma _{(0,1)} t)}},\qquad \end{aligned}$$

109 $$\begin{aligned} \nonumber \\&\tilde{x}_{02}(t|x_{(0,2)}(t_R))\nonumber \\&\quad = \frac{x_{(0,2)}(t_R)\exp {(\sigma _{(0,2)} t)}}{x_{(0,2)}(t_R)\exp {(\sigma _{(0,2)} t)}+1-x_{(0,2)}(t_R)-\frac{\bar{\nu }(t_R)}{N}+\frac{\bar{\nu }(t_R)}{N}\exp {(\sigma _{(0,1)} t)}}\qquad \end{aligned}$$

with $\bar{\nu }(t_R) = \frac{1}{p_{\text {est}}^{(0,1)}(x_{(0,2)})(t_R))}$. The time-dependent selection coefficient of type $(0,0)$ is given by

110 $$\begin{aligned} \tilde{s}_{(0,0)}(t|x_{(0,2)}(t_R)) = \sigma _{(0,0)} - \sigma _{(0,1)}\tilde{x}_{01}(t|x_{(0,2)}(t_R)) - \sigma _{(0,2)} \tilde{x}_{02}(t|x_{(0,2)}(t_R)).\nonumber \\ \end{aligned}$$

The establishment probability of a single individual of type $(0,0)$ that arises at time $t$ after $t_R$ can be calculated similarly to before and is given by

111 $$\begin{aligned} \tilde{p}^{(0,0)}_{\text {est}}(t|x_{(0,2)}(t_R))&= \frac{1}{A}\sigma _{(0,0)}(\sigma _{(0,0)}-\sigma _{(0,1)})(\sigma _{(0,0)}-\sigma _{(0,2)}),\nonumber \\ A&= (\sigma _{(0,0)}\!-\!\sigma _{(0,1)})(\sigma _{(0,0)}\!-\!\sigma _{(0,2)})\tilde{q}(t|x_{(0,2)}(t_R)) \nonumber \\&+\sigma _{(0,0)}(\sigma _{(0,0)}\!-\!\sigma _{(0,2)})\tilde{x}_{01}(t|x_{(0,2)}(t_R)) \nonumber \\&+ \sigma _{(0,0)} (\sigma _{(0,0)}\!-\!\sigma _{(0,1)})\tilde{x}_{02}(t|x_{(0,2)}(t_R)). \end{aligned}$$

The rate of successful type $(0,0)$ individuals is

112 $$\begin{aligned} \tilde{\alpha }_{(0,0)}(t|x_{(0,2)}(t_R))=Nr_{(0,0)}^{(0,1)}\tilde{q}(t|x_{(0,2)}(t_R))(1-\tilde{q}(t|x_{(0,2)}(t_R)))\tilde{p}^{(0,0)}_{\text {est}}(t|x_{(0,2)}(t_R)).\nonumber \\ \end{aligned}$$

(Remember: $r_{(0,0)}^{(0,1)}=r_{(0,0)}^{(0,2)}$.) We assume that we can ignore deviations from the deterministic frequency path for large $t$ close to fixation of the beneficial allele. The probability that no successful recombination event takes place is then given by

113 $$\begin{aligned} \text {Prob}(\text {no rec}|x_{(0,2)}(t_R)) \approx \exp {(-\int \limits _0^{\infty } \tilde{\alpha }_{(0,0)}(t|x_{(0,2)}(t_R)) \mathrm {d}t)}. \end{aligned}$$

The probability that type $(0,1)$ fixes is hence

114 $$\begin{aligned} P_{\text {det}}^{((0,2)\rightarrow (0,1))}&\approx \int \limits _0^{\infty } Q_{\text {det}}^{(0,0)}(x_{(0,2)}(t_R)) Q_{\text {det}}^{(0,1)}(x_{(0,2)}(t_R)) \alpha _{(0,1)}(t_R)\nonumber \\&\times \text {Prob}(\text {no rec}|x_{(0,2)}(t_R))\mathrm {d}t_R. \nonumber \\&= \frac{1}{\sigma _{(0,2)}}\int \limits _0^1 Q_{\text {det}}^{(0,0)}(x) Q_{\text {det}}^{(0,1)}(x) N r_{(0,1)}^{(0,2)} p_{\text {est}}^{(0,1)}(x) \text {Prob}(\text {no rec}|x)\mathrm {d}x.\nonumber \\ \end{aligned}$$

The overall probability that type $(0,0)$ fixes is obtained as

115 $$\begin{aligned} P_{\text {det}}^{((0,2)\rightarrow (0,0))}&\approx \frac{1}{\sigma _{(0,2)}}\int \limits _0^1 Q_{\text {det}}^{(0,0)}(x) Q_{\text {det}}^{(0,1)}(x) N r_{(0,0)}^{(0,2)} p_{\text {est}}^{(0,0)}(x)\mathrm {d}x\nonumber \\&\quad +\frac{1}{\sigma _{(0,2)}}\int \limits _0^1 Q_{\text {det}}^{(0,0)}(x) Q_{\text {det}}^{(0,1)}(x) N r_{(0,1)}^{(0,2)} p_{\text {est}}^{(0,1)}(x)\nonumber \\&\quad \times (1-\text {Prob}(\text {no rec}|x))\mathrm {d}x. \end{aligned}$$

### The process is initiated by an individual of type $(1,1)$

We now focus on four types $(0,0)$, $(0,1)$, $(1,0)$, and $(1,1)$, which all have a positive selection coefficient. An individual of type $(1,1)$ starts sweeping. We derive an approximation which is valid if no more than two successful recombination events happen during the process, i.e., for weak recombination. We again assume that recombination does not significantly reduce the number of offspring of any individual’s own type.

The deterministic path of type $(1,1)$ is given by

116 $$\begin{aligned} x_{(1,1)} = \frac{\bar{\nu }_0 \exp {(\sigma _{(1,1)} t)}}{N-\bar{\nu }_0+\bar{\nu }_0 \exp {(\sigma _{(1,1)} t)}} \end{aligned}$$

with $\bar{\nu }_0=\frac{1}{1-q_{(1,1)}}$, where $q_{(1,1)}$ is defined in Eq. (26) as $q_{(1,1)}=\frac{1-\sigma _{(1,1)}}{1-r_{(1,1)}}$. Recombination events that generate successful lineages occur at rate

117 $$\begin{aligned} \alpha _{(1,1)}(t) = \alpha _{(1,0)}(t)+\alpha _{(0,1)}(t) \end{aligned}$$

with

118a $$\begin{aligned} \alpha _{(1,0)}(t)&= N x_{(1,1)}(t)(1-x_{(1,1)}(t)) r_{(1,0)}^{(1,1)} p_{\text {est}}^{(1,0)}(x_{(1,1)}(t)), \end{aligned}$$

118b $$\begin{aligned} \alpha _{(0,1)}(t)&= Nx_{(1,1)}(t)(1-x_{(1,1)}(t))r^{(1,1)}_{(0,1)} p_{\text {est}}^{(0,1)}(x_{(1,1)}(t)), \end{aligned}$$

where $p_{\text {est}}^{(1,0)}$ and $p_{\text {est}}^{(0,1)}$ are the establishment probabilities of an individual of type $(1,0)$ and $(0,1)$ respectively and calculated by suitable substitutions in Eq. (38). Analogous to Eq. (41), the probability that no successful recombination event takes place is given by

119 $$\begin{aligned} P_{\text {det}}^{((1,1)\rightarrow (1,1))}&\approx \exp {\left[ -\int \limits _0^{\infty } \alpha _{(1,1)}(t)\mathrm {d}t\right] } = \left( \frac{\sigma _{(1,0)}}{\sigma _{(1,0)}-\sigma _{(1,1)}}\right) ^{\!\!-\frac{Nr^{(1,1)}_{(1,0)} \sigma _{(1,0)}(\sigma _{(1,0)}-\sigma _{(1,1)})}{\sigma _{(1,1)}^2}}\nonumber \\&\times \left( \frac{\sigma _{(0,1)}}{\sigma _{(0,1)}-\sigma _{(1,1)}}\right) ^{\!\!-\frac{Nr^{(1,1)}_{(0,1)} \sigma _{(0,1)}(\sigma _{(0,1)}-\sigma _{(1,1)})}{\sigma _{(1,1)}^2}}. \end{aligned}$$

The probability that no successful type $(1,0)$ is generated up to $t_R$ is given by

120 $$\begin{aligned}&\exp {\left[ -\int \limits _0^{t_R} \alpha _{(1,0)}(t) \mathrm {d}t\right] }\nonumber \\&\quad \approx \exp {\left[ -\!\!\!\!\! \int \limits _{0}^{x_{(1,1)}(t_R)} \!\!\!\!\! Nr^{(1,1)}_{(1,0)} \frac{1}{\sigma _{(1,1)}}\frac{\sigma _{(1,0)} (\sigma _{(1,0)}-\sigma _{(1,1)})}{ (\sigma _{(1,0)}-\sigma _{(1,1)})(1-x) + \sigma _{(1,0)}x} \mathrm {d}x\right] }\nonumber \\&\quad = \left( \frac{\sigma _{(1,0)} - (1 - x_{(1,1)}(t_R))\sigma _{(1,1)}}{\sigma _{(1,0)} - \sigma _{(1,1)}}\right) ^{-\frac{N r_{(1,0)}^{(1,1)}\sigma _{(1,0)}(\sigma _{(1,0)} - \sigma _{(1,1)})}{\sigma _{(1,1)}\sigma _{(1,1)}}} \equiv Q_{\text {det}}^{(1,0)}(x_{(1,1)}(t_R)).\nonumber \\ \end{aligned}$$

The probability that no successful type $(0,1)$ is generated up to $t_R$ is given by

121 $$\begin{aligned}&\exp {\left[ -\int \limits _0^{t_R} \alpha _{(0,1)}(t) \mathrm {d}t\right] } \nonumber \\&\quad \approx \exp {\left[ -\!\!\!\!\! \int \limits _{0}^{x_{(1,1)}(t_R)} \!\!\!\!\! Nr^{(1,1)}_{(0,1)} \frac{1}{\sigma _{(1,1)}}\frac{\sigma _{(0,1)} (\sigma _{(0,1)}-\sigma _{(1,1)})}{ (\sigma _{(0,1)}-\sigma _{(1,1)})(1-x) + \sigma _{(0,1)}x} \mathrm {d}x\right] }\nonumber \\&\quad = \left( \frac{\sigma _{(0,1)} - (1 - x_{(1,1)}(t_R))\sigma _{(1,1)}}{\sigma _{(0,1)} - \sigma _{(1,1)}}\right) ^{-\frac{N r_{(0,1)}^{(1,1)}\sigma _{(0,1)}(\sigma _{(0,1)} - \sigma _{(1,1)})}{\sigma _{(1,1)}\sigma _{(1,1)}}}\nonumber \\&\quad \equiv Q_{\text {det}}^{(0,1)}(x_{(1,1)}(t_R)). \end{aligned}$$

The probability that as a first event, a successful type $(1,0)$ individual is generated is given by

122 $$\begin{aligned}&\int \limits _0^{\infty } Q_{\text {det}}^{(1,0)}(x_{(1,1)}(t_R)) Q_{\text {det}}^{(0,1)}(x_{(1,1)}(t_R)) \alpha _{(1,0)}(t_R)\mathrm {d}t_R\nonumber \\&\quad =\int \limits _0^{\infty } Q_{\text {det}}^{(1,0)}(x_{(1,1)}(t_R)) Q_{\text {det}}^{(0,1)}(x_{(1,1)}(t_R))\nonumber \\&\qquad \times N x_{(1,1)}(t)(1-x_{(1,1)}(t)) r_{(1,0)}^{(1,1)} p_{\text {est}}^{(1,0)}(x_{(1,1)}(t)) \mathrm {d}t_R \nonumber \\&\quad = \frac{1}{\sigma _{(1,1)}}\int \limits _0^1 Q_{\text {det}}^{(1,0)}(x) Q_{\text {det}}^{(0,1)}(x) N r_{(1,0)}^{(1,1)} p_{\text {est}}^{(1,0)}(x)\mathrm {d}x. \end{aligned}$$

Equivalently, the probability that, as a first event, a successful type $(0,1)$ individual is generated is given by

123 $$\begin{aligned} \frac{1}{\sigma _{(1,1)}}\int \limits _0^1 Q_{\text {det}}^{(1,0)}(x) Q_{\text {det}}^{(0,1)}(x) N r_{(0,1)}^{(1,1)} p_{\text {est}}^{(0,1)}(x)\mathrm {d}x. \end{aligned}$$

We now choose without loss of generality $\sigma _{(1,0)}\le \sigma _{(0,1)}$.

We first consider the case that as a first event, an individual of type $(0,1)$ is generated. In that case, type $(0,1)$ might either rise to fixation, or a successful individual of type $(0,0)$ might be generated in the following process. We ignore the possibility that an individual of type $(1,0)$ might be generated and temporally sweep until it goes extinct. We now calculate the probability of the two events given a successful individual of type $(0,1)$ has been generated at time $t_R$. A recombination event may generate a successful type $(0,1)$ individual at time $t_R$. The type $(0,1)$ individual will rise to fixation unless a successful type $(0,0)$ individual is generated. In order to calculate this probability, we need the frequencies of the three present types (wildtype, type $(0,1)$, $(1,1)$) at time $t$ after the recombination event:

124a $$\begin{aligned}&\tilde{q}(t|x_{(1,1)}(t_R))\nonumber \\&\quad = \frac{1-x_{(1,1)}(t_R)-\frac{\bar{\nu }(t_R)}{N}}{x_{(1,1)}(t_R)\exp {(\sigma _{(1,1)} t)}+1-x_{(1,1)}(t_R)-\frac{\bar{\nu }(t_R)}{N}+\frac{\bar{\nu }(t_R)}{N}\exp {(\sigma _{(0,1)} t)}},\qquad \end{aligned}$$

124b $$\begin{aligned}&\tilde{x}_{01}(t|x_{(1,1)}(t_R))\nonumber \\&\quad = \frac{\frac{\bar{\nu }(t_R)}{N}\exp {(\sigma _{(0,1)} t)}}{x_{(1,1)}(t_R)\exp {(\sigma _{(1,1)} t)}+1-x_{(1,1)}(t_R)-\frac{\bar{\nu }(t_R)}{N}+\frac{\bar{\nu }(t_R)}{N}\exp {(\sigma _{(0,1)} t)}}, \qquad \end{aligned}$$

124c $$\begin{aligned}&\tilde{x}_{11}(t|x_{(1,1)}(t_R))\nonumber \\&\quad =\frac{x_{(1,1)}(t_R)\exp {(\sigma _{(1,1)} t)}}{x_{(1,1)}(t_R)\exp {(\sigma _{(1,1)}t)}+1-x_{(1,1)}(t_R)-\frac{\bar{\nu }(t_R)}{N}+\frac{\bar{\nu }(t_R)}{N}\exp {(\sigma _{(0,1)} t)}}\qquad \end{aligned}$$

with $\bar{\nu }(t_R) = \frac{1}{p_{\text {est}}^{(0,1)}(x_{(1,1)}(t_R))}$. The time-dependent selection coefficient of type $(0,0)$ is given by

125 $$\begin{aligned} \tilde{s}_{(0,0)}(t|x_{(1,1)}(t_R)) = \sigma _{(0,0)} - \sigma _{(0,1)}\tilde{x}_{01}(t|x_{(1,1)}(t_R)) - \sigma _{(1,1)} \tilde{x}_{11}(t|x_{(1,1)}(t_R)).\nonumber \\ \end{aligned}$$

The establishment probability of a type $(0,1)$ individual that arises at time $t$ after $t_R$ can be calculated as before and is given by

126 $$\begin{aligned} \tilde{p}^{(0,0)}_{\text {est}}(t|x_{(1,1)}(t_R))&= \frac{1}{A}\sigma _{(0,0)}(\sigma _{(0,0)}-\sigma _{(0,1)})(\sigma _{(0,0)}-\sigma _{(1,1)}),\nonumber \\ A&= (\sigma _{(0,0)}\!-\!\sigma _{(0,1)})(\sigma _{(0,0)}\!-\!\sigma _{(1,1)})\tilde{q}(t|x_{(1,1)}(t_R))\nonumber \\&+\,\,\sigma _{(0,0)}(\sigma _{(0,0)}\!-\!\sigma _{(1,1)})\tilde{x}_{01}(t|x_{(1,1)}(t_R)) \nonumber \\&+\,\,\sigma _{(0,0)} (\sigma _{(0,0)}\!-\!\sigma _{(0,1)})\tilde{x}_{11}(t|x_{(1,1)}(t_R)). \end{aligned}$$

The rate of successful type $(0,0)$ individuals is

127 $$\begin{aligned} \tilde{\alpha }_{(0,0)}(t|x_{(1,1)}(t_R))=Nr_{(0,0)}^{(0,1)}\tilde{q}(t|x_{(1,1)}(t_R))\tilde{x}_{01}(t|x_{(1,1)}(t_R))\tilde{p}^{(0,0)}_{\text {est}}(t|x_{(1,1)}(t_R)).\nonumber \\ \end{aligned}$$

The probability that no successful recombination event takes place is therefore given by

128 $$\begin{aligned} \text {Prob}(\text {no rec}|x_{(1,1)}(t_R)) \approx \exp {\left[ -\int \limits _0^{\infty } \tilde{\alpha }_{(0,0)}(t|x_{(1,1)}(t_R)) \mathrm {d}t\right] }. \end{aligned}$$

The probability that type $(0,1)$ fixes is therefore given by:

129 $$\begin{aligned} P_{\text {det}}^{((1,1)\rightarrow (0,1))}&\approx \int \limits _0^{\infty } Q_{\text {det}}^{(1,0)}(x_{(1,1)}(t_R)) Q_{\text {det}}^{(0,1)}(x_{(1,1)}(t_R)) \alpha _{(0,1)}(t_R)\nonumber \\&\text {Prob}(\text {no rec}|x_{(1,1)}(t_R))\mathrm {d}t_R.\nonumber \\&= \frac{1}{\sigma _{(1,1)}}\int \limits _0^1 Q_{\text {det}}^{(1,0)}(x) Q_{\text {det}}^{(0,1)}(x) N r_{(0,1)}^{(1,1)} p_{\text {est}}^{(0,1)}(x) \text {Prob}(\text {no rec}|x)\mathrm {d}x.\nonumber \\ \end{aligned}$$

Accordingly, the probability that type $(0,0)$ fixes (given a successful individual of type $(0,1)$ has been generated) is given by

130 $$\begin{aligned} \frac{1}{\sigma _{(1,1)}}\int \limits _0^1 Q_{\text {det}}^{(1,0)}(x) Q_{\text {det}}^{(0,1)}(x) N r_{(0,1)}^{(1,1)} p_{\text {est}}^{(0,1)}(x) (1-\text {Prob}(\text {no rec}|x))\mathrm {d}x. \end{aligned}$$

We now turn to the case that an individual of type $(1,0)$ is generated first. A recombination event may generate a successful type $(1,0)$ individual at time $t_R$. Type $(1,0)$ will rise to fixation unless a successful type $(0,0)$ or type $(0,1)$ individual is generated. In order to calculate these probabilities, we again need the frequencies of the three present types (wildtype, type $(1,0)$, $(1,1)$) at time $t$ after the recombination event:

131a $$\begin{aligned}&\tilde{Q}(t|x_{(1,1)}(t_R)) \nonumber \\&\quad = \frac{1-x_{(1,1)}(t_R)-\frac{\bar{\mu }(t_R)}{N}}{x_{(1,1)}(t_R)\exp {(\sigma _{(1,1)} t)}+1-x_{(1,1)}(t_R)-\frac{\bar{\mu }(t_R)}{N}+\frac{\bar{\mu }(t_R)}{N}\exp {(\sigma _{(1,0)} t)}}, \qquad \end{aligned}$$

131b $$\begin{aligned}&\tilde{X}_{10}(t|x_{(1,1)}(t_R)) \nonumber \\&\quad = \frac{\frac{\bar{\mu }(t_R)}{N}\exp {(\sigma _{(1,0)} t)}}{x_{(1,1)}(t_R)\exp {(\sigma _{(1,1)} t)}+1-x_{(1,1)}(t_R)-\frac{\bar{\mu }(t_R)}{N}+\frac{\bar{\mu }(t_R)}{N}\exp {(\sigma _{(1,0)} t)}}, \qquad \end{aligned}$$

131c $$\begin{aligned}&\tilde{X}_{11}(t|x_{(1,1)}(t_R)) \nonumber \\&\quad = \frac{x_{(1,1)}(t_R)\exp {(\sigma _{(1,1)} t)}}{x_{(1,1)}(t_R)\exp {(\sigma _{(1,1)} t)}+1-x_{(1,1)}(t_R)-\frac{\bar{\mu }(t_R)}{N}+\frac{\bar{\mu }(t_R)}{N}\exp {(\sigma _{(1,0)} t)}}\qquad \end{aligned}$$

with $\bar{\mu }(t_R) = \frac{1}{p_{\text {est}}^{(1,0)}(x_{(1,1)}(t_R))}$. The time-dependent selection coefficient of a type $(0,0)$ individual is given by

132 $$\begin{aligned} \tilde{S}_{(0,0)}(t|x_{(1,1)}(t_R)) = \sigma _{(0,0)} - \sigma _{(1,0)}\tilde{X}_{01}(t|x_{(1,1)}(t_R)) - \sigma _{(1,1)} \tilde{X}_{11}(t|x_{(1,1)}(t_R)).\nonumber \\ \end{aligned}$$

The time-dependent selection coefficient of a type $(0,1)$ individual is given by

133 $$\begin{aligned} \tilde{S}_{(0,1)}(t|x_{(1,1)}(t_R)) = \sigma _{(0,1)} - \sigma _{(1,0)}\tilde{X}_{10}(t|x_{(1,1)}(t_R)) - \sigma _{(1,1)} \tilde{X}_{11}(t|x_{(1,1)}(t_R)).\nonumber \\ \end{aligned}$$

The establishment probability of a type $(0,0)$ individual that arises at time $t$ after $t_R$ can be calculated as before and is given by

134 $$\begin{aligned} \tilde{\pi }_{\text {est}}^{(0,0)}(t|x_{(1,1)}(t_R))&= \frac{1}{A} \sigma _{(0,0)}(\sigma _{(0,0)}-\sigma _{(1,0)})(\sigma _{(0,0)}-\sigma _{(1,1)}),\nonumber \\ A&= (\sigma _{(0,0)}\!-\!\sigma _{(1,0)})(\sigma _{(0,0)}\!-\!\sigma _{(1,1)})\tilde{Q}(t|x_{(1,1)}(t_R)) \nonumber \\&+\,\, \sigma _{(0,0)}(\sigma _{(0,0)}\!-\!\sigma _{(1,1)})\tilde{X}_{10}(t|x_{(1,1)}(t_R)) \nonumber \\&+\,\, \sigma _{(0,0)} (\sigma _{(0,0)}\!-\!\sigma _{(1,0)})\tilde{X}_{11}(t|x_{(1,1)}(t_R)). \end{aligned}$$

and accordingly for the establishment probability of a type $(0,1)$ individual

135 $$\begin{aligned} \tilde{\pi }_{\text {est}}^{(0,1)}(t|x_{(1,1)}(t_R))&= \frac{1}{B}\sigma _{(0,1)}(\sigma _{(0,1)}-\sigma _{(1,0)})(\sigma _{(0,1)}-\sigma _{(1,1)}),\nonumber \\ B&= (\sigma _{(0,1)}\!-\!\sigma _{(1,0)})(\sigma _{(0,1)}\!-\!\sigma _{(1,1)})\tilde{Q}(t|x_{(1,1)}(t_R)) \nonumber \\&+\,\,\sigma _{(0,1)}(\sigma _{(0,1)}\!-\!\sigma _{(1,1)})\tilde{X}_{10}(t|x_{(1,1)}(t_R))\nonumber \\&+\,\, \sigma _{(0,1)} (\sigma _{(0,1)}\!-\!\sigma _{(1,0)})\tilde{X}_{11}(t|x_{(1,1)}(t_R)). \end{aligned}$$

The rate of successful type $(0,0)$ individuals is

136 $$\begin{aligned} \tilde{\beta }_{(0,0)}(t|x_{(1,1)}(t_R))=Nr_{(0,0)}^{(1,0)}\tilde{Q}(t|x_{(1,1)}(t_R))\tilde{X}_{10}(t|x_{(1,1)}(t_R))\tilde{\pi }_{\text {est}}^{(0,0)}(t|x_{(1,1)}(t_R)).\nonumber \\ \end{aligned}$$

The rate of successful type $(0,1)$ individuals is

137 $$\begin{aligned} \tilde{\beta }_{(0,1)}(t|x_{(1,1)}(t_R))=Nr^{(1,1)}_{(0,1)}\tilde{Q}(t|x_{(1,1)}(t_R))\tilde{X}_{11}(t|x_{(1,1)}(t_R))\tilde{\pi }_{\text {est}}^{(0,1)}(t|x_{(1,1)}(t_R)).\nonumber \\ \end{aligned}$$

The probability that no successful recombination event takes place is therefore given by

138 $$\begin{aligned} \text {Prob}_2(\text {no rec}|x_{(1,1)}(t_R))\approx \exp {\left[ -\int \limits _0^{\infty } \tilde{\beta }_{(0,0)}(t|x_{(1,1)}(t_R))+\tilde{\beta }_{(0,1)}(t|x_{(1,1)}(t_R)) \mathrm {d}t\right] }.\nonumber \\ \end{aligned}$$

The probability that type $(1,0)$ fixes is therefore given by:

139 $$\begin{aligned} P_{\text {det}}^{((1,1)\rightarrow (1,0))}&\approx \int \limits _0^{\infty } Q_{\text {det}}^{(1,0)}(x_{(1,1)}(t_R)) Q_{\text {det}}^{(0,1)}(x_{(1,1)}(t_R)) \alpha _{(1,0)}(t_R)\nonumber \\&\quad \times \text {Prob}_2(\text {no rec}|x_{(1,1)}(t_R))\mathrm {d}t_R.\nonumber \\&= \frac{1}{\sigma _{(1,1)}}\int \limits _0^1 Q_{\text {det}}^{(1,0)}(x) Q_{\text {det}}^{(0,1)}(x) N r_{(1,0)}^{(1,1)} p_{\text {est}}^{(1,0)}(x) \text {Prob}_2(\text {no rec}|x)\mathrm {d}x.\nonumber \\ \end{aligned}$$

The probability that a successful type $(0,0)$ individual is generated next at time $\tau _R$, is given by

140 $$\begin{aligned} \frac{1}{\sigma _{(1,1)}}\!\!\int \limits _0^1 Q_{\text {det}}^{(1,0)}(x) Q_{\text {det}}^{(0,1)}(x) N r_{(1,0)}^{(1,1)} p_{\text {est}}^{(1,0)}(x) \mathrm {e}^{-\int \limits _0^{\tau _R} \tilde{\beta }_{(0,0)}(t|x)+\tilde{\beta }_{(0,1)}(t|x)\mathrm {d}t} \tilde{\beta }_{(0,0)}(\tau _R|x) \mathrm {d}x.\nonumber \\ \end{aligned}$$

The probability that a successful type $(0,0)$ is generated next at any time is thus

141 $$\begin{aligned}&\frac{1}{\sigma _{(1,1)}}\int \limits _0^1 Q_{\text {det}}^{(1,0)}(x) Q_{\text {det}}^{(0,1)}(x) N r_{(1,0)}^{(1,1)} p_{\text {est}}^{(1,0)}(x) \nonumber \\&\quad \times \int \limits _0^{\infty } \mathrm {e}^{-\int \limits _0^{\tau _R} \tilde{\beta }_{(0,0)}(t|x)+\tilde{\beta }_{(0,1)}(t|x)\mathrm {d}t} \tilde{\beta }_{(0,0)}(\tau _R|x)\mathrm {d}\tau _R \mathrm {d}x. \end{aligned}$$

The probability that a successful type $(0,1)$ individual is generated next, is given by

142 $$\begin{aligned}&\frac{1}{\sigma _{(1,1)}}\int \limits _0^1 Q_{\text {det}}^{(1,0)}(x) Q_{\text {det}}^{(0,1)}(x) N r_{(1,0)}^{(1,1)} p_{\text {est}}^{(1,0)}(x)\nonumber \\&\quad \times \int \limits _{0}^{\infty }\mathrm {e}^{-\int \limits _0^{\tau _R} \tilde{\beta }_{(0,0)}(t|x)+ \tilde{\beta }_{(0,1)}(t|x)\mathrm {d}t}\tilde{\beta }_{(0,1)}(\tau _R|x) \mathrm {d}\tau _R \mathrm {d}x. \end{aligned}$$

As numerical evaluation of these integrals is computationally expensive, we introduce the following approximation: We calculate the probability that at least one individual of type $(0,0)$ is generated, ignoring type $(0,1)$ (and vice versa), i.e.,

$$\begin{aligned} \int \limits _0^{\infty } \exp {\left[ -\int \limits _0^{\tau _R} \tilde{\beta }_{(0,0)}(t|x)\mathrm {d}t\right] } \tilde{\beta }_{(0,0)}(\tau _R|x)\mathrm {d}\tau _R = 1-\exp {\left[ -\int \limits _0^{\infty } \tilde{\beta }_{(0,0)}(t|x)\mathrm {d}t\right] } \end{aligned}$$

instead of

$$\begin{aligned} \int \limits _0^{\infty } \mathrm {e}^{-\int \limits _0^{\tau _R} \tilde{\beta }_{(0,0)}(t|x) +\tilde{\beta }_{(0,1)}(t|x)\mathrm {d}t} \tilde{\beta }_{(0,0)}(\tau _R|x)\mathrm {d}\tau _R. \end{aligned}$$

With this approximation, we define

143 $$\begin{aligned} P_{(0,0)}^{\text {upper bound}}&= \frac{1}{\sigma _{(1,1)}}\int \limits _0^1 Q_{\text {det}}^{(1,0)}(x) Q_{\text {det}}^{(0,1)}(x) N r_{(1,0)}^{(1,1)} p_{\text {est}}^{(1,0)}(x) \nonumber \\&\times \left( 1-\exp {\left[ -\int \limits _0^{\infty } \tilde{\beta }_{(0,0)}(t|x)\mathrm {d}t\right] } \right) \mathrm {d}x \end{aligned}$$

and equivalently $$P_{(0,1)}^{\text {upper bound}}$$.

In order to make the fixation probabilities of the various types sum up to one, we subsequently introduce a normalization factor, which overall leads to the approximation

144 $$\begin{aligned} P_{(0,0)}^{\text {upper bound}} \times \frac{\frac{1}{\sigma _{(1,1)}}\int \limits _0^1 Q_{\text {det}}^{(1,0)}(x) Q_{\text {det}}^{(0,1)}(x) N r_{(1,0)}^{(1,1)} p_{\text {est}}^{(1,0)}(x) (1-\text {Prob}_2(\text {no rec}|x))\mathrm {d}x}{P_{(0,0)}^{\text {upper bound}}+P_{(0,1)}^{\text {upper bound}}}\nonumber \\ \end{aligned}$$

for the probability that type $(0,0)$ is generated next at any time.

For the probability that type $(0,1)$ is generated next at any time, we approximate accordingly

145 $$\begin{aligned} P_{(0,1)}^{\text {upper bound}} \times \frac{\frac{1}{\sigma _{(1,1)}}\int \limits _0^1 Q_{\text {det}}^{(1,0)}(x) Q_{\text {det}}^{(0,1)}(x) N r_{(1,0)}^{(1,1)} p_{\text {est}}^{(1,0)}(x) (1-\text {Prob}_2(\text {no rec}|x))\mathrm {d}x}{P_{(0,0)}^{\text {upper bound}}+P_{(0,1)}^{\text {upper bound}}}\nonumber \\ \end{aligned}$$

After establishment of type $(0,1)$, a successful individual of type $(0,0)$ might still be generated. We ignore this probability. This will be a good approximation when the probability of three successful recombination events is low, i.e., in particular when recombination is low.

The probability that type $(0,0)$ rises to fixation is then approximately given by the sum of Eq.  (130) and Eq.  (144). And the probability that type $(0,0)$ rises to fixation is approximately given by the sum of Eq. (129) and Eq.  (145).

## Appendix G: Diffusion with killing for $I=0$, $J=1$

Hartfield and Otto (2011) derive a diffusion equation for the probability that type $(0,1)$ fixes in the population conditioned on fixation of the beneficial allele. We here give the equation adjusted to our model (for the derivation, we refer to Hartfield and Otto (2011)). We define:

146a $$\begin{aligned} S_{(0,0)}&= N\sigma _{(0,0)}, \end{aligned}$$

146b $$\begin{aligned} S_{(0,1)}&= N\sigma _{(0,1)}, \end{aligned}$$

146c $$\begin{aligned} \rho ^{(0,1)}_{(0,0)}&= Nr_{(0,0)}^{(0,1)}. \end{aligned}$$

The scaled version of Eq. (38) reads:

147 $$\begin{aligned} \pi (p) = \frac{S_{(0,0)}(S_{(0,0)}-S_{(0,1)})}{(S_{(0,0)}-S_{(0,1)})(1-p) + S_{(0,0)}p}, \end{aligned}$$

where $p$ is the relative frequency of type $(0,1)$. The mean change in $p$ over a time step measured in $N$ generations is the same in both models. The variance in change of $p$ is $V(p)=2p(1-p)$ in our model (vs $V(p)=p(1-p)$ in the Wright-Fisher model). Following Hartfield and Otto (2011), we denote by $P^*(p)$ the conditional probability that type $(0,1)$ fixes if its current frequency is $p$. We obtain:

148 $$\begin{aligned} \frac{\mathrm {d}^2P^*(p)}{\mathrm {d}p^2} + \sigma _{(0,1)} \frac{1+e^{-pS_{(0,1)}}}{1-e^{-pS_{(0,1)}}} \frac{\mathrm {d}P^*(p)}{\mathrm {d}p} - \rho _{(0,0)}^{(0,1)} \pi (p) P^*(p)=0. \end{aligned}$$

The differential equation is solved with the boundary conditions

149a $$\begin{aligned} P^*(1)&= 1, \end{aligned}$$

149b $$\begin{aligned} \left. \frac{\mathrm {d}P^*(p)}{\mathrm {d}p}\right| _{p=0}&= 0. \end{aligned}$$

Figure 11 compares the results for the hitchhiking probability as obtained via Eq. (148) and via Eq. (53). Note that the diffusion equation is conditioned on establishment of type $(0,1)$, assuming an absolute rate of increase of $\sigma _{(0,1)}$, while $\bar{\nu }$ is based on an absolute rate of increase of $\sigma _{(0,1)}-r_{(0,1)}$.

## Appendix H: Accuracy of the approximations

### Appendix H.1: The hitchhiking probability: the stochastic phase

Our approximation (43) is based on the assumption that only one “rescue individual” is generated. Here, we investigate the limits of this assumption. As an example, we consider the case $I=0$, $J=2$ with $\sigma _{(0,2)}<0$ in more detail. We denote by $\tilde{h}_1(s_0,s_1)$ the joint p.g.f. for the number of “successful” ($Y_+$) and “unsuccessful” ($Y_-$) recombination events until the extinction of type $(0,2)$ conditioned on survival of the process. A successful recombination event means that the recombinant founds an infinite lineage. It holds:

150 $$\begin{aligned} \tilde{h}_1(s_0,s_1)&= \sum \limits _{k_1=0}^{\infty } \sum \limits _{k_2=0}^{\infty } P(Y_+=k_1, Y_-=k_2|\text {survival}) s_0^{k_1} s_1^{k_2}\nonumber \\&= \sum \limits _{k_1=0}^{\infty } \sum \limits _{k_2=0}^{\infty } \frac{P(Y_+=k_1, Y_-=k_2, \text {survival})}{1-Q_{(0,2)}} s_0^{k_1} s_1^{k_2}\nonumber \\&= \sum \limits _{k_1=0}^{\infty } \sum \limits _{k_2=0}^{\infty } \frac{P(Y_+=k_1, Y_-=k_2, \text {survival})}{1-Q_{(0,2)}} s_0^{k_1} s_1^{k_2}\nonumber \\&= \frac{h_1(s_0,s_1)}{1-Q_{(0,2)}} - \sum \limits _{k_2=0}^{\infty } \frac{P(Y_+=0,Y_-=k_2)}{1-Q_{(0,2)}}s_1^{k_2} \end{aligned}$$

with $h_1(s_0,s_1)$ given by Eq. (11) and Lemma 6. The probability that more than one successful recombination event takes place is given by

151 $$\begin{aligned} P(Y_+>1|\text {survival})&= 1-P(Y_+=1|\text {survival})\nonumber \\&= 1-\frac{\partial }{\partial s_1}\tilde{h}_1(s_1,s_2)|_{s_1=0,s_2=1}\nonumber \\&= 1-\frac{P_{\text {success}}}{1-Q_{(0,2)}}h^{\prime }(1-P_{\text {success}}). \end{aligned}$$

The smaller $P(Y_+>1|\text {survival})$, the better is the assumption fulfilled. Panels A and B of Fig. 12 show the probability $P(Y_+>1|\text {survival})$ as a function of recombination and fitness of type $(0,2)$, respectively. Note that $P(Y_+>1|\text {survival})$ displays a maximum as a function of the recombination probability (not shown): For low recombination, an offspring is a recombinant type with low probability. For large recombination, the number of offspring which are themselves of type $(0,2)$ gets significantly reduced by recombination, leading to a faster extinction of type $(0,2)$; this in turn leads to few recombination events even if the recombination probability is high. Panels C and D of Fig. 12 show the hitchhiking probabilities corresponding to the scenarios of Panels A and B. The deviation between the theory and simulations is smaller than expected from $P(Y_+>1|\text {survival})$. For $\sigma _{(0,2)}$ close to zero, type $(0,2)$ can survive for a long time until it goes extinct and $P(Y_+>1|\text {survival})$ is large. However, successful recombination events are rare. This implies that, within the multitype branching process, the time between the generation of the first and second rescue individuals can be large. Assume that a rescue individual of type $(0,1)$ is generated first. Until the generation of the second rescue individual, type $(0,1)$ has already reached such a high frequency that the multitype branching process description has become obsolete. I.e., even if the multitype branching process predicts the generation of a second rescue individual of type $(0,0)$, the fixation of type $(0,0)$ is not certain in the full model.

### Appendix H.2: The hitchhiking probability: the deterministic phase

Our results for $P_{\text {det}}^{(1,1)\rightarrow (0,0)}$ and $P_{\text {det}}^{(1,1)\rightarrow (0,1)}$ rely on the assumption that no more than two successful recombination events happen until fixation of the adaptive allele (and additionally on an approximation of two integrals, see Eqs. (144) and (145). This assumption is only justified if recombination is weak. In particular, if recombination is strong, it is likely that a lineage of type $(0,1)$ individuals and a lineage of type $(1,0)$ individuals segregate simultaneously in the population and recombine to found a successful lineage of type $(0,0)$ individuals. In this case, our approximation will strongly overestimate the fixation probability of type $(0,1)$ (assuming that $\sigma _{(0,1)}>\sigma _{(1,0)}$) and underestimate the fixation probability of type $(0,0)$. This can be clearly seen in Fig. 13. Note that for increasing recombination, the fixation probability of type $(0,1)$ attains a maximum and decreases then in favor of fixation of type $(0,0)$ due to the described mechanism (not shown). This behavior is not covered by our theory. The prediction for fixation of type $(1,0)$ or $(1,1)$ remains highly accurate though.

### Appendix H.3: The hitchhiking probability: concatenation of the stochastic and the deterministic phase

Our theory is based on a clear separation of the process into two phases. In some cases, however, the two phases blend together.

In Fig. 5, Panel d, deviations between theory and simulations arise for increasing recombination. This has two reasons: First, the approximation $P_{(0,2)}^{(0,2)}\approx 1$ becomes worse as recombination increases. Second (and more importantly here), it is not unlikely that type $(0,1)$ and type $(0,2)$ establish simultaneously while our approximation assumes that type $(0,1)$ establishes after type $(0,2)$. A slight shift in the time of establishment as introduced by our approach can lead to appreciable deviations of the modeled frequency paths of both types from their true paths. This, in turn, has a non-negligible impact on the assessment of the hitchhiking probability.
